# Comparative Analysis of *Mycobacterium tuberculosis pe* and *ppe* Genes Reveals High Sequence Variation and an Apparent Absence of Selective Constraints

**DOI:** 10.1371/journal.pone.0030593

**Published:** 2012-04-04

**Authors:** Christopher R. E. McEvoy, Ruben Cloete, Borna Müller, Anita C. Schürch, Paul D. van Helden, Sebastien Gagneux, Robin M. Warren, Nicolaas C. Gey van Pittius

**Affiliations:** 1 Department of Science and Technology, National Research Foundation Centre of Excellence in Biomedical Tuberculosis Research, Medical Research Council Centre for Molecular and Cellular Biology, Stellenbosch University, Tygerberg, Cape Town, South Africa; 2 Tuberculosis Reference Laboratory, National Institute for Public Health and the Environment, Centre for Infectious Disease Control, (CIb/LIS, pb 22), Bilthoven, The Netherlands; 3 Department of Medical Parasitology and Infection Biology, Swiss Tropical and Public Health Institute, Basel, Switzerland; 4 University of Basel, Basel, Switzerland; 5 Division of Mycobacterial Research, Medical Research Council, National Institute for Medical Research, London, United Kingdom; Institut de Pharmacologie et de Biologie Structurale, France

## Abstract

*Mycobacterium tuberculosis* complex (MTBC) genomes contain 2 large gene families termed *pe* and *ppe*. The function of pe/ppe proteins remains enigmatic but studies suggest that they are secreted or cell surface associated and are involved in bacterial virulence. Previous studies have also shown that some *pe*/*ppe* genes are polymorphic, a finding that suggests involvement in antigenic variation. Using comparative sequence analysis of 18 publicly available MTBC whole genome sequences, we have performed alignments of 33 *pe* (excluding *pe_pgrs*) and 66 *ppe* genes in order to detect the frequency and nature of genetic variation. This work has been supplemented by whole gene sequencing of 14 *pe*/*ppe* (including 5 *pe_pgrs*) genes in a cohort of 40 diverse and well defined clinical isolates covering all the main lineages of the *M. tuberculosis* phylogenetic tree. We show that nsSNP's in *pe* (excluding *pgrs*) and *ppe* genes are 3.0 and 3.3 times higher than in non-*pe*/*ppe* genes respectively and that numerous other mutation types are also present at a high frequency. It has previously been shown that non-*pe*/*ppe M. tuberculosis* genes display a remarkably low level of purifying selection. Here, we also show that compared to these genes those of the *pe*/*ppe* families show a further reduction of selection pressure that suggests neutral evolution. This is inconsistent with the positive selection pressure of “classical” antigenic variation. Finally, by analyzing such a large number of genes we were able to detect large differences in mutation type and frequency between both individual genes and gene sub-families. The high variation rates and absence of selective constraints provides valuable insights into potential *pe/ppe* function. Since pe/ppe proteins are highly antigenic and have been studied as potential vaccine components these results should also prove informative for aspects of *M. tuberculosis* vaccine design.

## Introduction

*Mycobacterium tuberculosis*, the main causative agent of tuberculosis in humans, is a member of the *M. tuberculosis* complex (MTBC), a closely related group of slow-growing pathogenic mycobacteria. Recent studies of MTBC evolution have revealed that the *M. tuberculosis* genome appears to be a composite genome created by frequent horizontal gene transfer events in a broad, genetically diverse, progenitor species prior to an evolutionary bottleneck or selective sweep around 35,000 years ago [1]. Divergence of the rare, smooth colony forming tubercle bacilli *M. canetti* seems to immediately predate this bottleneck/selective sweep while all other members of the MTBC are the result of the clonal expansion of a small number of surviving bacteria. This recent clonal expansion with the concurrent absence of horizontal gene transfer explains the relatively high degree of genetic homogeneity (99.9%) observed between MTBC members despite differences in their phenotypic characteristics and host ranges [2], [3], [4]. Whole genome sequencing of several *M. tuberculosis* strains, along with *M. bovis* and *M. africanum*, has confirmed this genetic homogeneity and revealed many other interesting biological aspects [5], [6], [7].

One of the surprises emerging from the analysis of the first sequenced *M. tuberculosis* genome (the laboratory strain H37Rv) was the discovery of two large gene families, designated *pe* and *ppe*, that in H37Rv comprise 99 and 69 members respectively and together account for around 10% of the organism's genomic coding potential [5]. *Pe* genes are characterised by the presence of a proline-glutamic acid (PE) motif at positions 8 and 9 within a highly conserved N-terminal domain consisting of around 110 amino acids. Similarly, *ppe* genes contain a proline-proline-glutamic acid (ppe) at positions 7–9 in a highly conserved N-terminal domain of approximately 180 amino acids. The C-terminal domains of both pe and ppe protein families are highly variable in both size and sequence and often contain repetitive DNA sequences that differ in copy number between genes [5].

**Figure 1:**
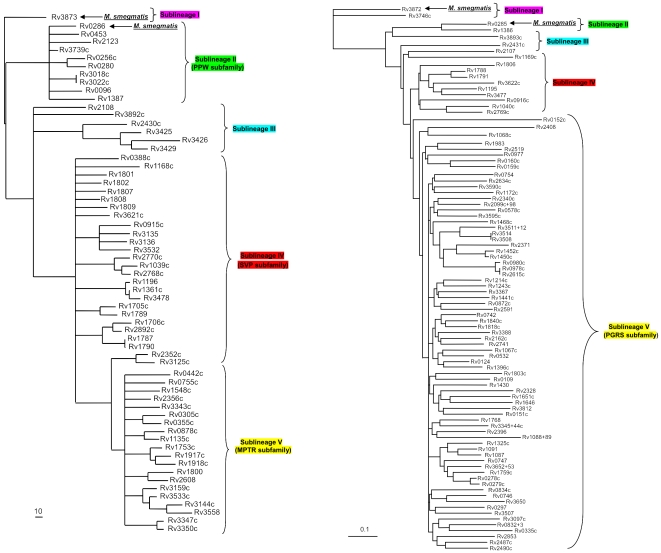
Phylogenetic reconstruction of the evolutionary relationships between the members of the pe and ppe protein families. A. Phylogeny of the ppe protein family. The phylogenetic tree was constructed from the phylogenetic analysis done on the 180 aa N-terminal domains of the ppe proteins. The tree was rooted to the outgroup Rv3873 (ppe68), shown to be the first ppe insertion into the ESAT-6 (esx) gene clusters [8]. Figure reproduced from reference 8 with permission of the authors. B. Phylogeny of the pe protein family. The phylogenetic tree was constructed from the phylogenetic analysis done on the 110 aa N-terminal domains of the pe proteins. The tree was rooted to the outgroup Rv3872 (pe35), shown to be the first pe insertion into the ESAT-6 (esx) gene clusters [8]. Figure reproduced from reference 8 with permission of the authors.

The *pe* and *ppe* gene families can be divided into sub-families based on similarities in their N-terminal regions and the phylogenetic relationships between each gene sub-family have been previously described, demonstrating that their evolutionary expansions are linked to the duplications of the *ESAT-6* (*esx*) gene clusters [8]. *Ppe* genes can be subdivided into 5 subfamilies, the most numerous of which are the *ppe_svp* (24 members) and the *ppe_mptr* (major polymorphic tandem repeat) subfamilies (23 members) (Fig. 1a). *Pe* genes can also be divided into 5 sub-families, the largest of which, the polymorphic GC-rich-repetitive sequence (*pe_pgrs*), comprises 65 members in H37Rv (Fig. 1b). This sub-family is characterised by a C-terminal domain that contains multiple tandem repeats of a glycine-glycine-alanine (Gly-Gly-Ala) or a glycine-glycine-asparagine (Gly-Gly-Asn) motif. Phylogenetic analysis indicates that the emergence of the large *pe_pgrs* and *ppe_mptr* subfamilies is a recent evolutionary event, with their presence being restricted to members of the MTBC and close relatives such as *M. marinum* and *M. ulcerans* [8].

The high *pe*/*ppe* gene content of the MTBC suggests an important biological role for their respective proteins. However, their precise function is unknown although recent studies have provided some intriguing clues. In pathogenic organisms it is generally found that proteins that are directly exposed to host immune surveillance show higher levels of polymorphism than that found in general housekeeping proteins [9]. This is thought to reflect their in volvement in antigenic variation and immune evasion. Many pe/ppe proteins have been found to be highly immunogenic and several groups have investigated this aspect of their biology with regard to vaccine production (for example, [10], [11]). Persuasive evidence now exists that many *M. tuberculosis* pe/ppe proteins are cell surface located [12], [13], [14], [15] and that others are probably secreted [16], [17] and this, in conjunction with their immunogenicity and the well established polymorphic nature of their C-terminal repeats, has led to the suggestion that they may well be involved in antigenic variation and immune evasion [5]. Indeed, several studies have revealed varying degrees of *pe*/*ppe* sequence polymorphism between *M. tuberculosis* clinical isolates. Talarico and colleagues have reported a high degree of polymorphism within the *pe_pgrs33*, *pe_pgrs16* and *pe_pgrs26* genes [18], [19], [20]. Similar results have also been found for *ppe18* [21]. Less sensitive analysis based on the size of DNA repeats in the C-terminal region of *ppe34* and *ppe8* have also revealed a high frequency of polymorphism [12], [22]. In addition, some *pe/ppe* genes have been reported to display elevated levels of IS*6110* integration [23], [24], [25], [26], [27], [28] and homologous recombination [26], [29], [30], [31]. However, sequence analysis of 4 *pe* (*pe5*, *pe11*, *pe18* and *pe31*) and 4 *ppe* (*ppe9*, *ppe27*, *ppe41* and *ppe50*) genes found polymorphism to be limited or absent [32]. Along with sequence variation, gene expression alterations may contribute to antigenic variation and these have also been noted in *pe*/*ppe* genes from different *M. tuberculosis* strains [33], [34], [35]. For example, *ppe44* shows limited sequence diversity between strains (only isolates of the Beijing genotype were found to be polymorphic) whereas transcript levels of the gene are highly variable [35]. Numerous other reports have documented variation in *pe*/*ppe* transcription levels under different environmental and experimental conditions [36], [37], [38], [39]. Furthermore, there does not appear to be a global regulator of *pe*/*ppe* expression [36], [40], suggesting a complex regulatory network and a high degree of plasticity in their expression repertoire.

It has been proposed that pe/ppe proteins can aid *M. tuberculosis* pathogenesis by negatively influencing host immunity [5] and recently Toll-like receptor 2 (TLR2) has assumed a prominent role in this theory. For example, Basu et al showed that pe_pgrs33 is able to enhance the expression of tumour necrosis factor alpha (TNFα) in a TLR2-dependent manner leading to macrophage apoptosis [41]. Interestingly, deletions within the PGRS domain (as is often seen in clinical isolates) inhibited this ability. Ppe proteins have also been shown to function in a TLR2-dependent manner. Nair et al demonstrated that ppe18 binds to TLR2 which stimulates IL-10 production in macrophages [42]. This leads to an anti-inflammatory Th2 type immune response. Evidence also exists to suggest that pe_pgrs proteins may be able to inhibit antigen processing and/or presentation [43] and it has been proposed that the Gly-Ala repeats in the C-terminal PGRS domains are able to inhibit proteasomal degradation of the N-terminal PE domain [44] thus inhibiting antigen processing by CD8+ T cells in a manner similar to that seen in Epstein – Barr virus nuclear antigen 1 [45]. Several other lines of evidence also suggest a major role for pe/ppe proteins in mycobacterial pathogenesis. For example, recent work has shown that pe_pgrs33 localises to host cell mitochondria where it is able to induce apoptosis and primary necrosis [46]. Studies demonstrating increased mycobacterial growth in macrophages and subsequent macrophage necrosis of pe_pgrs33 expressing strains (as oppose to pe_pgrs33 negative strains) have also been reported [47], [48] and other studies have documented an attenuated phenotype with the knockout of specific *pe*/*ppe* genes [49], [50] or the upregulation of specific *pe*/*ppe* genes upon infection [38], [51].

Evidence for other diverse alternative or additional pe/ppe functions also exists. *In silico* analysis of PGRS protein sequences reveal that at least 56 pe_pgrs members contain multiple nona-peptide repeats (GGXGXD/NXUX, where X = any amino acid and U = a large non-polar hydrophobic residue) that are predicted to be calcium binding motifs [52]. The authors suggest that these motifs might be involved in the initial attachment of *M. tuberculosis* to host alveolar macrophages. PGRS domains have also been implicated in cellular structure and colony morphology [14] and in the binding of fibronectin [53]. A possible role in iron uptake has also been proposed for ppe37 following the finding that it is upregulated under low iron conditions [54]. It is also notable that *pe*/*ppe* genes are often found paired within operons with the *pe* gene located upstream of the *ppe* gene. *Pe*/*ppe* genes within these operons are cotranscribed and physically interact with each other and transcription of both is required for correct cellular localization [55], [56]. This is emphasised by the findings of Strong and colleagues who failed in numerous attempts to determine the crystal structures of individual pe and ppe proteins. Coexpression and copurification of the proteins coded by the linked genes Rv*2431c* (*pe25*) and Rv*2430c* (*ppe41*) was successful, however, and the crystal structure revealed a 1∶1 pe25/ppe41 protein dimer where helices from each protein are predicted to interact and form a stable complex. The structure implies a docking site for an additional protein and suggests a role in signal transduction [56].

Here, we have used recently acquired whole genome sequence data from 18 isolates representing a broad spectrum of the MTBC phylogeny to investigate variation in 33 *pe* (excluding *pe_pgrs*) and 66 *ppe* genes. We have supplemented this data by selecting 14 *pe* and *ppe* genes (including 5 *pe_pgrs*) and performing whole gene sequencing on a cohort of 40 clinical isolates representing a broad and well characterised spectrum of the *M. tuberculosis* phylogeny. We hypothesise that if pe/ppe proteins are involved in immune evasion and antigenic variation their genes will have undergone rapid evolutionary change, as demonstrated by high levels of DNA sequence polymorphism and evidence for diversifying selection compared to other *M. tuberculosis* genes. Previous work on this theme [12], [18], [19], [20], [21], [22], [32] has produced conflicting results that may be due to the lack of sensitivity of the analysis technique, the decision to examine genes that belong to a particular *pe* or *ppe* sub-family that might show abnormal variation levels, or the decision to examine clinical isolates that are too closely related to reveal polymorphic differences. The resultant comparative gene analysis presented here provides new insights into the variation and evolution of these genes along with their potential role in providing the pathogen with a source of antigenic variation.

## Results

### Comparative gene analysis using whole genome sequences

A total of 66 *ppe* and 33 *pe* genes were analysed. Unfortunately, due to the extensive repetitiveness of their C-terminal regions and the inherent difficulties encountered in sequencing through repetitive regions using the third generation short read sequencing techniques, the *pe_pgrs* genes of most publicly available whole genome sequences were incomplete or of low sequence quality and could not be included in this analysis. Variability estimates for *ppe38*/*71* and *ppe50* could not be determined due to the difficulty in obtaining a reference sequence. *Ppe38*/*71* are completely homologous in most cases and are located in a hypervariable region that is prone to homologous recombination, gene conversion, IS*6110* integration and large deletion events [26]. *Ppe50* is also highly variable and displays numerous different sequence types due to large deletions and other sequence variations [32]. Due to the exclusion of genes with notations suggesting potential sequence errors, an average of 15.2 and 16.5 genomes (from a possible maximum of 18) were analysed for each *ppe* and *pe* gene respectively. Full details of all variations detected can be seen in tables S1, S2, S3.

### Confirmation of whole genome sequence accuracy

In order to ascertain the accuracy of the whole genome sequences used in our analysis we obtained the original DNA used in the sequencing process to determine the F11, CPHL_A, K85, T17 and T92 sequences. A total of 40 variations observed in the *pe/ppe* genes of these 5 isolates were reanalysed by amplifying the surrounding region by PCR and using standard Sanger sequencing methodology to sequence the amplicons. A variety of variations were chosen for analysis and these comprised sSNP's, nsSNP's, frameshifts, and an in-frame deletion. We also ensured that some of the variations detected in the large *ppe_mptr* genes, *ppe5*/*6* and *ppe7*/*8*, were analysed since it could be suggested that mistakes are more likely to be made here due to the highly repetitive nature of their C-terminal domains. Table S4 lists the variations, primer details and results of our analysis. Four of the 40 variations (10%) were found to be erroneous in the publicly available whole genome sequences. One of these (T17, *ppe28*) appears to be due to an assembly error while another (CPHL_A, *ppe13*) involves a long poly C region at the 3′ end of the gene. The other 2 errors involve a SNP or single bp deletion. The 10 variations that were checked in the *ppe5*/*6* and *7*/*8* genes were all confirmed indicating that the large *ppe_mptr* genes were not more likely to produce sequencing errors than the smaller less complex genes.

### Number of structural protein variants

**Figure 2:**
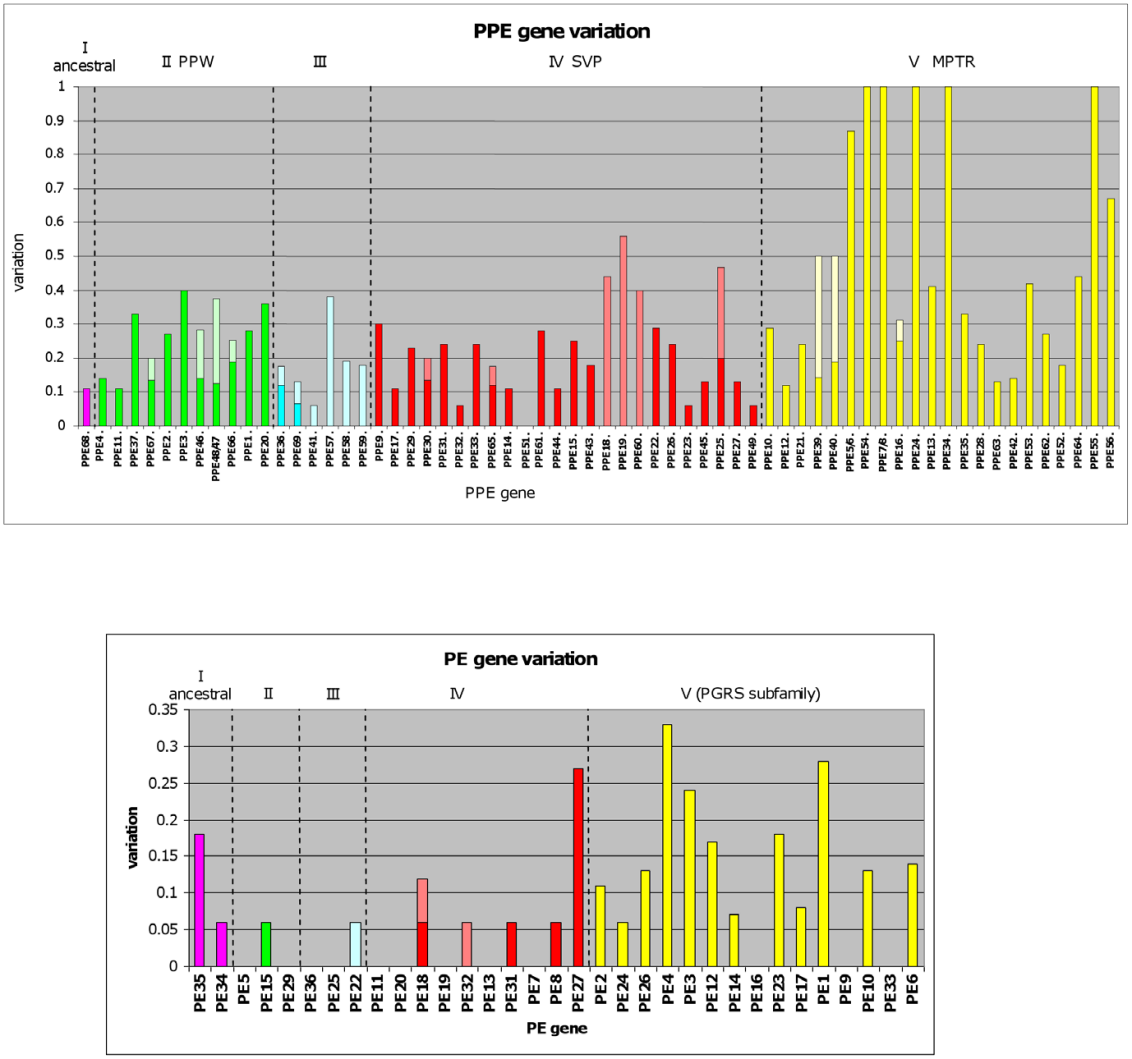
Sequence variation levels in *ppe* and *pe* genes. A. Calculations of sequence variation in 64 *ppe* genes. Synonymous variations have been ignored. The Y axis shows the proportion of sequences that show variation predicted to result in amino acid changes. A value of 1 indicates that all analysed sequences were unique. Average number of genomes analysed per gene = 15.2. Genes have been grouped together according to their subfamily [8] by colour and subfamilies are also separated by dotted lines. Each vertical bar is subdivided into micromutations (nsSNP's, frameshifts, small in-frame indels) in dark shading and macromutations (homologous recombination, IS6110 integration, partial and whole gene deletions) in light shading. *Ppe38* and *ppe50* were not included due to hypervariability at the macromutational level [26], [30] and the difficulty in establishing a consensus sequence. For details of all variations detected see Tables S1 and S2. B. Calculations of sequence variation in 33 *pe* (excluding *pgrs*) genes. Synonymous variations have been ignored. Average number of isolates analysed per gene = 16.5. The genes from subfamily V (*pgrs* subfamily, yellow) are those which are classified as members of this subfamily by their N-terminal amino acid sequences [8] but that do not include the long PGRS C-terminal region. For details of all variations detected see Tables S1 and S3.

Various aspects of genetic variation between the homologous genes may be analysed. First we wished to determine the number of predicted different structural variants of each pe/ppe protein, based on the observed genetic variations, as a proportion of the total number of isolates analysed. Thus, sSNP's were ignored, variations that were specific to multiple isolates from a single lineage were counted as a single variant and single isolates that contained more than 1 variation were still counted as a single variant. Results for the *ppe* gene analysis are shown in Fig. 2a. They reveal a high level of variation across all subfamilies, with only one gene (*ppe51*) showing no variation in all genomes analysed. Subfamily V (the MPTR subfamily) shows many genes with extreme levels of variation. By distinguishing between different types of mutation it is notable that certain genes display alternate mechanisms of variation. For example, homologous recombination events, particularly between closely related *ppe* genes in close physical proximity, are shown to be responsible for a high degree of variation within certain genes (*ppe57*/*58*/*59* and *ppe18*/*19*/*60*). Other macromutational events (whole or partial gene deletions and IS*6110* integrations) were found to be responsible for a significant proportion of the variation in several genes. Also notable is the finding that macromutational events do not contribute to variation in the most hypervariable genes of the MPTR subfamily. Six genes in particular (*ppe5/6*, *7/8*, *24*, *34*, *54* and *55*) show extreme variation and 5 of these have a variation index of 1 (indicating that each isolate had a unique sequence). These 6 genes are all large (between approximately 3.1 and 10.0 kb) and reveal mutations including nsSNP's, frameshifts and in-frame indels. The sequences of these 6 hypervariable genes were further compared between 3 closely related genomes, KZN 1435, KZN 605 and KZN 4207 [57] in order to ascertain whether they were evolving at a rate that would enable us to distinguish even between extremely closely related isolates. For each gene the sequence in all 3 genomes was identical. The sequences of four of the hypervariable *ppe* genes (*ppe24*, *34*, *54* and *55*) were also compared between the index case and 2 transmission chain endpoint isolates of the Harlingen cluster [58], [59]. An average of 84% of the coding region for each of these genes was available for analysis. No variations were observed. These results indicate that while these *ppe* genes are hypervariable across the full phylogeny of *M. tuberculosis*, they do not evolve at a rate fast enough to distinguish between extremely closely related isolates.

A similar analysis of the *ppe* (excluding *pe_pgrs*) genes revealed agenerally lower level of variation with many of the genes showing no variation across the analysed genomes (Fig. 2b). Macro-mutational events, including homologous recombination, were rare.

### *Pe*/*ppe* variation levels in comparison with other *M. tuberculosis* genes

In order to ascertain whether the variation levels of the *pe* and *ppe* genes differed from other *M. tuberculosis* genes we compared our results to those obtained by Hershberg and colleagues who identified the SNP's present in 89 non-*pe*/*ppe* genes comprising 65,829 bp, from 107 MTBC isolates [60]. This study identified a total of 231 nsSNP's, which when divided by the total number of nucleotides multiplied by the number of isolates gives a nsSNP frequency of 231/(65,829×107) = 0.327×10^−4^ nsSNP's per nucleotide. Similar calculations using our ppe data required the exclusion of several genes. *Ppe38*/*71* and *ppe50*, which exhibit extreme levels of macro-mutational variation, were excluded. *Ppe18*, *19*, *24*, *34*, *54*, *55*, *57*, *58*, *59* and *60* were also excluded because of extreme variability or frequent homologous recombination events which resulted in difficulty in determining the consensus sequence of the gene. The remaining 54 *ppe* genes comprise 100,657 bp and contain 163 nsSNP's and the average number of isolates analysed per gene was 15.17. This results in a nsSNP frequency of 163/(100,657×15.17) = 1.067×10^−4^ nsSNP's per nucleotide. This value is approximately 3.3-fold greater than that found in the non-*pe*/*ppe* MTBC genes [60] despite the exclusion of the most variable *ppe* genes. Similarly, the 33 *pe* (excluding *pgrs*) genes comprised 21,726 bp and contained 35 nsSNP's with an average isolate number per gene of 16.5, resulting in a nsSNP frequency of 35/(21,726×16.5) = 0.976×10^−4^ nsSNP's per nucleotide. This value is approximately 3.0-fold higher than that found in non-*pe*/*ppe* MTBC genes [60]. These results confirm that nsSNP's occur at a far higher frequency in *pe*/*ppe* genes than in non-*pe*/*ppe* genes.

### Whole gene sequencing results of 14 *pe* and *ppe* genes

Complete results for all variations found from whole gene sequencing of 14 *pe* and *ppe* genes from 40 phylogenetically diverse clinical isolates covering the whole *M. tuberculosis* phylogenetic tree (PGG1, 2 and 3 strains including members of all the main lineages EAI, CAS, Beijing, LAM, Haarlem, LCC and T) are shown in Table S5. Our sequencing of 3 *pe* genes (*pe35*, *11* and *3*) and 6 *ppe* genes (*ppe68*, *2*, *44*, *10*, *42* and *62*) confirmed the results found for these genes in our *in silico* gene analysis. In each case lineage specific variations were consistent between the 2 different analyses. Interestingly, our 3 EAI samples failed to show the *ppe62* G1690A SNP which was present in 2 (T17 and T46) of the 4 EAI samples analysed *in silico*. This suggests that our EAI isolates are relatively closely related and do not reflect the large genetic diversity observed within this group [60].

Particular interest lies in our analysis of the 5 *pe_pgrs* genes since the *pgrs* subfamily could not be analysed using *in silico* methods. Three of these genes (*pe_pgrs16*, *26* and *33*) have previously been analysed for their variation [18], [19], [20]. Our replication of this work (using better defined *M. tuberculosis* lineages) confirms that all 3 of these genes display extremely high variation with in-frame indels within the *pgrs* repetitive region comprising a large proportion of the mutations in each case. These indels were often large. For example, in the *pe_pgrs16* gene the 2 EAI isolates SAWC1659 and SAWC2493 both possess 2 deletions of 66 bp and 600 bp and all CAS family isolates possess 2 deletions of 45 bp and 42 bp in *pe_pgrs33* (Table S5). *Pe_pgrs18* has previously been reported as being part of a duplicated gene pair (with *pe_pgrs17*) that shows evidence of homologous recombination and gene conversion events [30]. Our results for *pe_pgrs18* confirmed a high level of homologous recombination with *pe_pgrs17*. We were also able to confirm the presence of the 12/40 polymorphism in the Haarlem and LCC groups that appears to result from gene conversion with *pe_pgrs17* [30]. Details of the variability characteristics of *pe_pgrs62* have not previously been reported. Results for this gene were surprising because despite being the same size as most of the other analysed *pe_pgrs* genes it showed very little variation and no in-frame indels were observed (Table S5). Additional *in silico* analysis of this gene in *M. bovis* confirmed its invariant nature. A closer inspection of the gene's predicted amino acid sequence revealed that it does not possess the C-terminal multiple tandem repeats of Gly-Gly-Ala and Gly-Gly-Asn typical of pgrs proteins. Taken together, our results suggest that *pe_pgrs* genes generally display high variation levels with in-frame indels making a large proportion of mutations. However, mutational mechanisms and levels of variation can differ greatly between individual genes implying functional variation within this subfamily.

The 252 bp deletion identified in the *pe_pgrs16* gene of isolate SAWC 2185 (Haarlem, F2) (Table S5) was further analysed in order to determine how variable this mutation was within both the F2 family and other members of the same cluster as SAWC 2185. Isolates from 36 different F2 clusters as well as 4 isolates from the same cluster were examined. All other members of the cluster to which isolate SAWC 2185 belongs, along with 27 of the additional F2 clusters, were found to contain this mutation. However, isolates representing the remaining F2 clusters lacked the mutation confirming the presence of within-family variation for this mutation.

### Analysis of selective constraints in *pe*/*ppe* genes

One of the major findings of the MTBC genetic diversity study of Hershberg and colleagues [60] was the low level of purifying selection compared to other bacteria, as assessed by the ratio of nonsynonymous to synonymous SNP's (dN/dS) in 89 non-*pe*/*ppe* genes. A dN/dS ratio of <1 is considered to indicate purifying selection, dN/dS = 1 suggests an absence of selection (i.e. neutral evolution) and dN/dS>1 indicates positive or diversifying selection. In our analysis of 54 *ppe* genes (excluding the genes described above) we discovered a total of 220 SNP's, of which 163 (74%) were nonsynonymous (Table S2). The average pairwise dN/dS ratio for the concatenated *ppe* genes was 1.045. This is substantially higher than the already extremely high value of 0.57 reported for the non-*pe*/*ppe M. tuberculosis* genes [60] and suggests an absence of selection pressure. Similarly, in our analysis of 33 *pe* genes we detected a total of 47 SNPs, of which 35 (74%) were nonsynonymous (Table S3). The average pairwise dN/dS ratio for the concatenated *pe* genes was 1.000, again far higher than the value previously obtained for non-*pe/ppe* genes [60] and again suggesting an absence of selection pressure. We also calculated the dN/dS value for the 3 *pe_pgrs* genes that show a “typical” pgrs variation profile (*pe_pgrs16*, *26* and *33* – see above) using the SNPs identified in our current study in addition to those detected previously [18], [19], [20]. A total of 63 SNPs were found in these 3 genes of which 43 (68%) were nonsynonymous. The average pairwise dN/dS ratio for the 3 concatenated *pe_pgrs* genes was 0.869, a value once again close to that indicating neutral evolution.

## Discussion

Although polymorphisms in certain *M. tuberculosis pe* and *ppe* genes have been previously documented, this study is the first to make use of publicly available MTBC whole genome sequences, as well as a comprehensive set of 40 clinical isolates covering the known *M. tuberculosis* phylogenetic tree and all major *M. tuberculosis* strain lineages including EAI, CAS, Beijing, Haarlem, LAM, LCC and T, to produce an extensive analysis of *pe/ppe* gene variation. Unfortunately, the large *pe_pgrs* subfamily was not able to be analysed using these methods due to a lack of sequencing accuracy but our own sequencing analysis of selected *pe_pgrs* genes, in conjunction with those of previous investigators, also provides important insights into genetic variation within this subfamily.

The first important observation made was the confirmation that *pe* and *ppe* genes display a high frequency of variation (Fig. 2) and that this variation exceeds that seen in other MTBC genes. Hershberg and colleagues previously analysed MTBC genetic diversity by sequencing 89 non-*pe/ppe* genes (classified as either “housekeeping”, “virulence” or “surface”) in 107 MTBC isolates [60]. Compared to these genes, the nsSNP frequency in the *ppe* and *pe* (excluding *pe_pgrs*) genes in our analysis was approximately 3.3 and 3.0 times greater, respectively. Several qualifying points should be emphasised when considering these values. Our quality assurance analysis of 40 selected SNP's from the whole genome sequences revealed that 4 were incorrect (Table S4), indicating an overestimation of variation frequency of approximately 10%. However, it should also be noted that our *ppe* variation values were obtained without the inclusion of the 12 most variable *ppe* genes which were excluded from the analysis due to the difficulty in determining a consensus sequence. The inclusion of these genes would undoubtedly result in a significant increase in observed *ppe* variation. In addition, many of the *ppe* genes that were included in our analysis displayed high levels of variation that were not due to nsSNP's. Indeed, the mutational spectrum seen in *ppe* genes was extensive. The most common mutations observed were nsSNP's and frameshifts caused by small indels. However, many large *ppe* genes of the *mptr* subfamily often display in-frame indels, certain groups of genes undergo frequent homologous recombination events and, as previously reported, *ppe39* and *ppe40* are particularly susceptible to IS*6110* insertions [27]. Figure 2a reveals that members of the *ppe_mptr* subfamily have a generally higher frequency of mutation but this does not apply consistently to all members of this group.

Interestingly, although the *pe* (excluding *pgrs*) genes revealed far lower variation than the *ppe's* (Fig. 2), the frequency of nsSNP's was similar to that of the *ppe's* and was found to be approximately triple that of the non-*pe*/*ppe* genes analysed previously [60]. Protein changes in these genes were generally due to nsSNP's and small indels leading to frame shifts. In-frame indels and macromutations (whole or partial gene deletions and IS*6110* integrations) were rare. The lower variation in these genes probably reflects a strong functional constraint of the pe protein. It has previously been shown that pe proteins and the pe domain of pe_pgrs proteins are responsible for cell wall localisation [15]. This is presumably essential for optimal protein function and mutations that hinder this process would therefore be subject to strong negative selection pressures.

Our own sequencing analysis of 5 *pe_pgrs* genes showed that, in general, variation within the *pe_pgrs* subfamily exists at far higher levels than in non-*pgrs pe* members and that this increase in variation is largely caused by a higher frequency of in-frame indels within the C-terminal *pgrs* region. These results support the findings of Talarico and colleagues who have previously reported analysis of genetic polymorphism in *pe_pgrs33*, *16* and *26* [18], [19], [20]. We show that the deletions in these 3 genes are often large (for example, in EAI isolates 666 bp has been deleted from *pe_pgrs16*, Table S5). The fact that large deletions were often found in multiple isolates from the same lineage suggests that these mutations are not subjected to strong purifying selection forces. The phenotypic consequences of these deletions may include a reduction in macrophage apoptosis caused by a decrease in TNFα production [41] and, at an epidemiological and clinical level, be associated with clustering and a lack of lung cavitations [19]. Our analysis of *pe_pgrs18* and *62* has provided additional interesting information relating to *pe_pgrs* variation since neither of these genes displayed the “typical” variation pattern seen in *pe_pgrs33*, *16* and *26* (Table S5). Variation in *pe_pgrs18* was found to be largely caused by gene conversion with *pe_pgrs17*. These genes are in close physical proximity, have high sequence homology, and are presumably the result of a recent duplication event. A previous study has documented homologous recombination between these genes and has identified a polymorphism present in one or both of these genes and used it to infer details of the evolution and clonal expansion of the MTBC [30]. Genetic variation in *pe_pgrs62* has not been reported previously and we chose this gene for analysis because studies have shown that it is a T cell antigen with vaccine potential [61], [62], [63] and that its PGRS domain is able to elicit a strong antibody response [64]. The PGRS domain of *pe_pgrs62* is atypical as it lacks the Gly-Gly-Ala or Gly-Gly-Asn repeats found in most members of this subfamily. Interestingly, the amount of variation seen in this gene was far less than in the more typical *pe_pgrs* genes with only 4 SNPs (of which only 2 were non-synonymous) seen in our 40 clinical isolates. This lack of genetic variation is especially interesting since *pe_pgrs62* can stimulate both cell-mediated and humoral based host immunity and might therefore be expected to undergo significant levels of antigenic variation. Taken together, these results reveal that while variation in *pe_pgrs* genes is generally very high, this variation, along with the dominant type of mutational mechanism, can differ greatly between genes. The finding of low variation in a highly immunogenic *pe_pgrs* member lacking the typical PGRS domain also implies functional variation in certain members of this sub-family.

Another major finding of this study was that selection appears to be absent in *pe/ppe* genes. Most genomic regions in all organisms are subjected to strong purifying selection pressures. Within the Actinobacteria, for example, pairwise genome-wide comparisons result in a general dN/dS value of 0.15–0.20 [60]. This value appears to be fairly typical of both prokaryotic and eukaryotic organisms [65]. The recent comparative sequence analysis of 89 genes in 107 MTBC isolates [60] found an average pairwise dN/dS ratio of 0.57, a value far higher than that found in other bacteria and an indication that purifying selection is severely reduced in the MTBC on a general genomic level. In *pe/ppe* genes specifically, a high ratio of nonsynonymous to synonymous SNP's has previously been noted [6], [20] and it has also been shown that these genes are under greater selection for amino acid substitutions than other *M. tuberculosis* genes [66]. Our pairwise dN/dS ratio calculations for 54 *ppe* and 33 *pe* genes were 1.045 and 1.000 respectively, suggesting that selection pressure on these genes is extremely limited or altogether absent. Although our analysis of *pe_pgrs* genes was numerically limited, the pairwise dN/dS ratio was also close to 1 (0.869), again indicating a selection pressure close to neutral. This result is surprising because pe/ppe proteins are thought to provide antigenic variation and therefore be subjected to positive, rather than neutral, selection pressure. Thus, our results indicate that variation in these proteins is inconsistent with “classical” antigenic variation. It should be noted, however, that these results are an average of the gene families as a whole and that individual genes might be subjected to greater or lesser selective pressures. Evidence that *pe/ppe* genes are the major targets of positive selection in *M. tuberculosis* comes from a recent paper that examined the genomes of H37Rv and H37Ra [67]. Of the 12 genes that were found to be positively selected in these strains 6 were from the *pe* or *pe_pgrs* families. Our results also need to be interpreted in light of the report of Comas and colleagues [68] who found that the antigenic epitopes (excluding pe and ppe proteins) of *M. tuberculosis* are highly conserved and that there appears to be a strong selection pressure against sequence diversity in these regions. This finding was unexpected and is also inconsistent with the classical model of an evolutionary immunological arms race between pathogen and host and the authors favour the explanation that the host immune response is, paradoxically, beneficial to the pathogen. Despite the fact that our *pe* and *ppe* dN/dS values were far higher than those found for *M. tuberculosis* antigens in the Comas study, this explanation may also apply (to a lesser extent) to pe and ppe proteins and explain why their dN/dS values were less than those of typical antigens in other organisms.

Although our results suggest that pe/ppe proteins do not act as typical antigenic variants it is also important to consider the impact of population genetics on dN/dS values. The dN/dS ratio is a popular measure of selection pressure not only because it is simple and robust but also because of the simple interpretation of dN/dS<1 as negative selection, dN/dS = 1 as neutral selection and dN/dS>1 as positive selection. This analysis was originally designed for comparisons between sequences from divergent lineages or species and it has recently been shown that the standard signature of positive selection (dN/dS>1) does not hold for comparisons within a population [69]. It can sometimes be difficult to determine the appropriate evolutionary time-scale (distinct lineages/species versus numerous isolates from a single population) associated with a dataset of microbial sequences and it is possible that some of the more closely related sequences in our dataset have not diverged sufficiently for this analysis to be appropriate. If this is the case it is unlikely that our dN/dS values would alter drastically. We would, however, predict that our values are underestimates and therefore conclude that a mild positive selection pressure is acting upon these genes. Many of the *pe/ppe* genes present within the MTBC have homologues in the closely related, but phylogenetically distinct, species *M. marinum* [70] and we suggest that a comparison between these genes could provide a more accurate estimate of the evolutionary pressures they have been subjected to.

We hope that our results will allow for a more directed approach towards the use of pe/ppe proteins as vaccine components since it is possible that the high levels of polymorphism observed in certain members of these protein families could limit their effectiveness in some cases. This has been highlighted in a recent mathematical modelling analysis that has predicted the negative impact on vaccine efficacy that may occur when mycobacterial strain diversity is not considered [71]. For example, the Mtb72F vaccine comprises the 2 recombinant proteins pepA (Rv0125) and ppe18 (Rv1196). Mtb72F has been shown to have a protective effect against challenges with two *M. tuberculosis* laboratory reference strains (H37Rv and Erdmann) in numerous animal models, including a primate model [72], [73], [74], [75]. However, a recent study has shown that over 20% of *M. tuberculosis* strains taken from 2 geographical regions contain mutations that alter at least 1 amino acid in the ppe18 protein, many of which are in regions predicted to be T cell epitopes [21]. Our study confirms a high rate of *ppe18* variation and shows that it is predominantly due to homologous recombination between *ppe18*, *ppe19* and *ppe60*, which have extremely high sequence similarity. These results suggest that the Mtb72F vaccine could have limitations in a clinical setting and that, in hindsight, a pe/ppe protein that displays higher sequence conservation across many strains may have been a more effective vaccine candidate. An example of this is pe_pgrs62 which has also been investigated for its vaccine potential with promising results [61], [62]. This highly immunogenic, atypical pgrs protein showed extremely limited sequence variation across our cohort of isolates (Table S5) and might be expected to provide more consistent protection against a variety of *M. tuberculosis* strains. The data available for immunogenicity at the *pe/ppe* epitope level is limited however and it should be noted that variable regions of *pe/ppe* genes may be less immunogenic and less important for an immune response. It should also be noted that pe/ppe proteins probably have functional variation and that some may have a limited role in immune function.

The exact nature of pe/ppe function in the host cell is yet to be determined. However, our results also provide some additional insights and allow us to speculate on potential mechanisms of action for these proteins. When the high levels of pe/ppe sequence variation are considered in conjunction with the high inter-strain expressional variation [33], [34], [35] it is apparent that there is likely to be a huge diversity of pe/ppe expressional and functional variation across the MTBC. This would lead to a situation where only extremely closely related isolates have identical functional and expressional profiles across the entire pe/ppe spectrum. We note that this situation has parallels to the classical MHC class I and II systems where highly polymorphic MHC loci produce multiple alleles which, despite their structural and functional similarities, are distinct with regards to the antigenic peptides they present to CD4+ and 8+ T cells. It may be speculated that the large number of polymorphic pe/ppe proteins have evolved in response to the multiple MHC alleles expressed by host populations and that specific pe/ppe proteins are adapted to preferentially coexist alongside specific MHC alleles. The absence of selection exerted on *pe/ppe* genes may be interpreted as both a result of immune pressure selecting for antigenic variants and an adaptation for these proteins to function alongside new or rare MHC alleles that have not previously been encountered in the bacteria's evolutionary history. Although purely speculative, this theory is consistent with the large *pe/ppe* expansion within the MTBC (and in the closely related species *M. marinum* which is a natural pathogen of fish), it's functional and expressional variability, and the finding that some pe/ppe proteins appear to interfere with antigen processing [43], [44]. The true nature of pe/ppe function remains one of the great mysteries of *M. tuberculosis* pathogenesis however and many additional functional studies will probably be required before we are able to gain a more complete understanding of their role.

## Materials and Methods

### Ethics statement

We recovered sputum specimens from the National Health Laboratory Service (NHLS) after routine processing. None of the authors were directly involved in sputum collection. This study was approved by the Stellenbosch University Health Research Ethics Committee (approval reference number N10/04/126). Informed consent was not required as we received samples from the NHLS after routine processing. This was approved by the Stellenbosch University IRB.

### *In silico* whole genome sequence analysis

#### Sequence selection details

Analysis of *pe* and *ppe* genes from the following 18 fully sequenced MTBC genomes was conducted: *M. bovis* strain AF2122/97 [76], H37RV [77], CDC1551 [6], CPHL_A (*M. africanum*), K85 (*M. africanum*), T92, T46, T48, EAS054, 94_M4241A, 02_1987, T85, C strain, Haarlem, F11, GM1503, KZN 1435 and 98-R604_INH-RIF-EM [57]. Details of the phylogenetic placements of each isolate are shown in Table 1. At least 1 representative from all 7 major MTBC lineages (including the animal lineage) [78] are included in this study apart from lineage 3 (CAS lineage). *Ppe* genes from the fully sequenced isolates KZN 605 and KZN 4207 [57] as well as the Harlingen transmission chain [58] were also analysed in specific instances. Orthologues of each gene were located by BLAST searches using the H37Rv gene sequence as the type standard. Gene sequences obtained from the Broad institute [57] were not used if they contained the following messages suggesting possible low sequence quality: “At least one base has a quality score <10”, “EST-based feature contains predicted/unverified ORF” or “Frame Shift: Sequence Error”. Sequence alignments were done using CLUSTALW [79].

**Table 1:** Details of 18 whole genome sequence isolates used for *in silico* comparative gene analysis.

Isolate	Lineage	Reference
T92	Lineage 1. PGG1, EAI family	[57]
T17	Lineage 1. PGG1, EAI family	[57]
T46	Lineage 1. PGG1, EAI family	[57]
EAS054	Lineage 1. PGG1, EAI family	[57]
94_M4241A	Lineage 2. PGG1, Beijing family	[57]
02_1987	Lineage 2. PGG1, Beijing family	[57]
T85	Lineage 2. PGG1, Beijing family	[57]
C strain	Lineage 4. PGG2, low copy clade	[57]
CDC1551	Lineage 4. PGG2, low copy clade	[6]
Haarlem	Lineage 4. PGG2, Haarlem family	[57]
F11	Lineage 4. PGG2, LAM family	[57]
GM1503	Lineage 4. PGG2, LAM family	[57]
KZN1435	Lineage 4. PGG2, LAM family	[57]
98-R604_INH-RIF-EM	Lineage 4. PGG2, LAM family	[57]
H37Rv	Lineage 4. PGG3	[5], [77]
CPHL_A	Lineage 5. PGG1, West Africa-1 (*M. africanum*)	[57]
K85	Lineage 6. PGG1, West Africa-2 (*M. africanum*)	[57]
*M. bovis* AF2122/97	Animal lineage	[76]

Each analysed genome sequence is listed along with its lineage number [78], Principal Genetic Group (PGG) [2] and family group.

#### Confirmation of whole genome sequence accuracy

Genomic DNA from 5 of the whole genome sequenced isolates (F11, CPHL_A, K85, T17 and T92) was used to check the accuracy of 40 variations that were found in various *pe* and *ppe* genes (Table S4). Primers were designed to amplify a region surrounding the variation point and PCRs and sequencing of the amplicons were performed as described below. Recently, a number of nucleotides in the H37Rv sequence, including some within *pe* and *ppe* genes, were found to be incorrect [78]. These SNPs were corrected before analysis.

#### dN/dS values

Due to the general low level of SNPs present when analysing individual genes, a concatenated alignment for each gene category (*ppe*, *pe* and *pe_pgrs*) was generated combining all individual genes. Prior to concatenation the consensus sequence of each gene was aligned with the equivalent sequence containing all SNP's identified using CLUSTALW [79]. Other variations (eg frameshifts or in-frame indels) that had been identified were ignored. The resultant alignment files for each gene were concatenated using DnaSP [80] and pairwise dN/dS values were determined by subjecting the alignment to the DnaSP program package.

### DNA sequencing of clinical isolates

#### Bacterial culture conditions, molecular typing and strain selection

Sputum samples were obtained from primary health care clinics in metropolitan Cape Town, South Africa. This region has a very high tuberculosis incidence and has been used extensively in an ongoing, prospective epidemiological study [81]. According to the National Tuberculosis Control Program in line with the Directly Observed Therapy Short-course strategy, diagnosis of tuberculosis is made by sputum smear microscopy in new cases, and by smear microscopy and culture in retreatment cases. We recovered these sputum specimens for our study area of interest from the National Health Laboratory Service (NHLS) after routine processing. *M. tuberculosis* strains present in sputum culture were genotyped using IS*6110* RFLP [82] and spoligotyping [83], [84]. DNA fingerprints were analysed with GelCompar software using the unweighted-pair group method, average linkages and Dice coefficients [85]. Isolates with an IS*6110* similarity index of ≥65% were grouped into strain lineages [86]. Fourty isolates of divergent lineages were selected for analysis. Table 2 shows phylogenetic details of these clinical isolates.

**Table 2:** Details of clinical isolates used in this study.

Isolate	Lineage	South African IS*6110* Lineage [84]
SAWC 1659	1, PGG1, EAI	-
SAWC 2493	1, PGG1, EAI	-
SAWC 4981	1, PGG1, EAI	-
SAWC 2803	3, PGG1, CAS	F34
SAWC 2240	3, PGG1, CAS	F20
SAWC 2666	3, PGG1, CAS	F33
SAWC 974	3, PGG1, CAS	F25
SAWC 2088	2, PGG1, Atypical Beijing	F31
SAWC 2701	2, PGG1, Atypical Beijing	F27
SAWC 2076	2, PGG1, Typical Beijing	F29
SAWC 1430	4, PGG2	F3
SAWC 3656	4, PGG2, LAM	F26
SAWC 2576	4, PGG2, LAM	F15
SAWC 2525	4, PGG2, LAM	F9
SAWC 1815	4, PGG2, LAM	F11
SAWC 1733	4, PGG2, LAM	F13
SAWC 3100	4, PGG2, LAM	F14
SAWC 1595	4, PGG2, Quebec/S	F28
SAWC 198	4, PGG2, “1 bander”	F110
SAWC 2073	4, PGG2, LCC – “2 bander”	F120
SAWC 233	4, PGG2, LCC – “3 bander”	F130
SAWC 861	4, PGG2, LCC – “4 bander”	F140
SAWC 1162	4, PGG2, LCC – “5 bander”	F150
SAWC 716	4, PGG2, Pre-Haarlem	F19
SAWC 1748	4, PGG2, Pre-Haarlem	F24
SAWC 1127	4, PGG2, Haarlem-like	F6
SAWC 103	4, PGG2, Haarlem-like	F7
SAWC 386	4, PGG2, Haarlem	F1
SAWC 1645	4, PGG2, Haarlem	F10
SAWC 1841	4, PGG2, Haarlem	F4
SAWC 2185	4, PGG2, Haarlem	F2
SAWC 239	4, PGG3, T	F22
SAWC 2901	4, PGG3, T	F16
SAWC 1608	4, PGG3, T	F5
SAWC 1109	4, PGG3, T	F23
SAWC 4302	4, PGG3, T	F18
SAWC 1956	4, PGG3, T	F17
SAWC 1290	4, PGG3, T	F21
SAWC 300	4, PGG3, T	F12
SAWC 1870	4, PGG3, T	F8

Each clinical isolate along with its lineage number [78], PGG group [2], spoligotype family group status [88] and South African IS*6110* lineage [84] is listed.

#### Selection of *pe*/*ppe* genes for whole gene sequence analysis

A phylogenetic analysis of both the *pe* and *ppe* gene families has previously been reported [8]. This has demonstrated that each gene family can be divided into several subfamilies (Fig. 1). In order to maximise the scope of our analysis we selected genes representative of several different sub-families in each case. Where possible, genes for which some aspect of their biology (such as antigenicity) had been previously reported were chosen. A total of 14 *pe/ppe* genes were selected. These included the ancestral member of each family as well as 5 *pe_pgrs* genes. Details of the selected genes are listed in Table 3.

**Table 3:** Details of the *pe* and *ppe* genes examined by whole gene sequencing.

Gene	Rv number	Size in H37Rv (bp)	Sublineage*	Variability in literature	Comments
*pe35*	Rv*3872*	300	I	No data.	Ancestral pe protein. Present in RD1 region. Highly immunogenic, eg [89].
*pe11*, *lipX*	Rv*1169c*	303	IV	Invariable [32].	B cell responses in subgroups of patients [90]. Putative lipase [77].
*pe3*	Rv*0159c*	1407	V (PGRS subfamily)	No data.	Atypical sublineage V protein. Not pgrs.
*pe_pgrs16*	Rv*0977*	2772	V (PGRS subfamily)	Highly variable [20].	Upregulated in mouse model [39], [91].
*pe_pgrs18*	Rv*0980c*	1374	V (PGRS subfamily)	Known to undergo homologous recombination with *pe_pgrs*17 [30].	Highly upregulated during the early stages of *M. tuberculosis* invasion of the blood-brain barrier [91]. High sequence identity to *pe_pgrs*17 implying recent duplication event [30].
*pe_pgrs26*	Rv*1441c*	1476	V (PGRS subfamily)	Highly variable [20].	Downregulated in mouse model [39].
*pe_pgrs33*	Rv*1818c*	1497	V (PGRS subfamily)	Highly variable [18], [19].	Localised in cell wall [14] and surface exposed [15]. SigA-mediated transcription downregulated during stationary phase and under stress conditions [92]. Implicated in pathogenicity and host immune responses [43], [47], [48], [61], [62], [90], [93], [94]. Possible inhibitor of antigen processing [44].
*pe_pgrs62*	Rv*3812*	1515	V (PGRS subfamily)	No data.	Elicits strong antibody response [64]. T cell antigen [61], [62].
*ppe68*	Rv*3873*	1107	I	No data.	Ancestral ppe protein.
*ppe2*	Rv*0256c*	1671	II (PPW subfamily)	No data.	PPW subfamily.
*ppe44*	Rv*2770c*	1149	IV (SVP subfamily)	Limited diversity. Alteration in Beijing isolates [35].	Variable expression in clinical isolates [35]. Expressed during subcutaneous and intravenous infection by *M. bovis* BCG in BALB/c mice [95].
*ppe10*	Rv*0442c*	1464	V (MPTR subfamily)	No data.	Ancestral ppe MPTR protein.
*ppe42*	Rv*2608*	1743	V (MPTR subfamily)	Variable in clinical isolates [96].	Elicits a high humoral and low T cell response [96].
*ppe62*	Rv*3533c*	1749	V (MPTR subfamily)	No data.	MPTR protein.

* **As defined in reference** [**8**] **.**

Each gene sequenced in this study is listed along with its phylogenetic position within its family and any additional information regarding its protein's function available in the literature.

#### PCR and sequencing

Primer sequences for the 14 selected genes are listed in Table S6. PCRs were done in a reaction mixture containing 0.1 µg template DNA, 3 µl GC-rich solution, 1.5 µl 10× buffer containing MgCl_2_, 2.4 µl 10 mM dNTP's, 0.6 µl each primer (5 pmol/µl) and 0.12 µl FastStart Taq (Roche, Germany) made up to 15 µl with H_2_O. Amplification comprised an initial 6 min template denaturation followed by 35 cycles using the appropriate annealing temperature (listed in Table S6) and an extension time of 30 s to 1 min 30 s depending on the length of the amplicon. PCR product was checked by electrophoresis through an agarose gel and an aliquot was treated with ExoSAP-IT (USB). Sequencing was performed using an ABI 3100 automated DNA sequencer. Sequence editing and manipulation was done using the BioEdit Sequence Alignment Editor [87].

## Supporting Information

Table S1 (DOCX)

Table S2 **2 homologous genes (PPE38 & PPE71) in most isolates. Hotspot for IS6110 integration and homologous recombination** [**26**] **.**
(XLS)

Table S3 (XLS)

Table S4 (DOCX)

Table S5 (DOCX)

Table S6 (DOCX)
