# Supplementary material for: Comparative Analysis of Mycobacterium tuberculosis pe and ppe Genes Reveals High Sequence Variation and an Apparent Absence of Selective Constraints
Source: PLoS One. 2012 Apr 4;7(4):e30593. doi: 10.1371/journal.pone.0030593 (PMC3319526; doi:10.1371/journal.pone.0030593)
Supplement: Table S1 — (DOCX) [file pone.0030593.s001.docx]

**Table S1.**

| **Gene** | **Sequence**  **variation** | **Position** | | **Genetic**  **change** | | | **Amino acid**  **change** | | **Number**  **of isolates** | | **Comments** | |
| --- | --- | --- | --- | --- | --- | --- | --- | --- | --- | --- | --- | --- |
| ***ppe68***  **Sublineage I**  18 isolates  1107 bp | **nsSNPs** | | | | | | | | | | | |
|  | nsS1 | 86 | | C → T | | | Ala → Val | | 1 | | Haarlem | |
|  | nsS2 | 685 | | G → C | | | Val → Leu | | 4 | | EAI specific. | |
| ***ppe4***  **Sublineage II (PPW subfamily)**  14 isolates  1542 bp | **Frameshift** | | | | | | | | | | | |
|  | FS1 | 1057 | | 1 bp insertion | | | Premature stop | | 1 | | 02_1987 | |
|  | **nsSNP** | | | | | | | | | | | |
|  | nsS1 | 941 | | T → C | | | Val → Ala | | 1 | | EAS054 | |
|  | **sSNP** |  | |  | | |  | |  | |  | |
|  | sS1 | 460 | | C → T | | | Val | | 1 | | K85. Confirmed. | |
|  | sS2 | 555 | | C → G | | | Ala | | 8 | | TBD1- specific. | |
| ***ppe11***  **Sublineage II (PPW subfamily)**  18 isolates  1557 bp | **nsSNP** | | | | | | | | | | | |
|  | nsS1 | 1288 | | C → T | | | Arg → Cys | | 3 | | CDC1551, strain C and Haarlem. | |
|  | nsS2 | 1510 | | A → G | | | Met → Val | | 1 | | F11. Confirmed. | |
|  | **sSNP** | | | | | | | | | | | |
|  | sS1 | 1248 | | G → A | | | Val | | 1 | | Strain C | |
| ***ppe37* Sublineage II (PPW subfamily)**  15 isolates  1422 bp | **In-frame deletion** | | | | | | | | | | | |
|  | D1 | 91 - 117 | | 27 bp deletion | | |  | | 1 | | *M. bovis* | |
|  | **Frameshifts** | | | | | | | | | | | |
|  | FS1 | 507 | | 1 bp deletion | | | Premature stop | | 2 | | Beijing isolates T85 and 02_1987. | |
|  | FS2 | 1017 - 1018 | | 2 bp deletion | | | No premature stop. | | 4 | | LAM specific. Incorrect amino  acid incorporation from codon 340. | |
|  | **nsSNPs** | | | | | | | | | | | |
|  | nsS1 | 370 | | G → A | | | Val → Met | | 1 | | CPHL_A Confirmed. | |
|  | nsS2 | 449 | | C → G | | | Ala → Gly | | 1 | | T85 | |
|  | nsS3 | 563 | | G → T | | | Ser → Ile | | 1 | | *M. bovis* | |
| ***ppe67***  **Sublineage II (PPW subfamily)**  15 isolates  234 bp | **Whole gene deletion** | | | | | | | | | | | |
|  | WGD1 | 1166bp deletion deletes *ppe67* and N-terminus of *ppe66*. | | | | | | | 1 | | *M. bovis.* | |
|  | **nsSNPs** | | | | | | | | | | | |
|  | nsS1 | 53 | | T → G | | | Leu → Arg | | 1 | | CPHL_A | |
|  | nsS2 | 233 | | A → G | | | Stop → Trp | | 2 | | PGG1 isolates T92 and CPHL_A.  Mutation adds additional 333 amino  acids before next stop codon.  Apparent convergent mutation. | |
|  | **sSNP** | | | | | | | | | | | |
|  | sS1 | 186 | | T → C | | | Gly | | 1 | | Haarlem | |
| ***ppe2***  **Sublineage II (PPW subfamily)**  11 isolates  1671 bp | **nsSNPs** | | | | | | | | | | | |
|  | nsS1 | 419 | | A → G | | | Glu → Gly | | 1 | | CPHL_A. Confirmed. | |
|  | nsS2 | 1211 | | C → T | | | Pro → Leu | | 1 | | CDC1551 | |
|  | nsS3 | 1292 | | G → A | | | Asp → Asn | | 1 | | CPHL_A. Confirmed. | |
|  | nsS4 | 1381 | | G → T | | | Ala → Ser | | 1 | | CDC1551 | |
|  | nsS5 | 1487 | | G → A | | | Gly → Asp | | 1 | | 94_M4241A | |
|  | **sSNP** | | | | | | | | | | | |
|  | sS1 | 1236 | | C → T | | | Thr | | 1 | | T46 | |
| ***ppe3***  **Sublineage II (PPW subfamily)**  10 isolates  1611 bp | **nsSNPs** | | | | | | | | | | | |
|  | nsS1 | 556 | | G → T | | | Asp → Tyr | | 1 | | T46 | |
|  | nsS2 | 769 | | G → A | | | Glu → Lys | | 2 | | CPHL_A and 02_1987. Apparent  convergent mutation. | |
|  | nsS3 | 1009 | | C → T | | | Pro → Ser | | 1 | | H37Rv | |
|  | nsS4 | 1344 | | G → T | | | Glu → Asp | | 1 | | CPHL_A | |
|  | **sSNPs** | | | | | | | | | | | |
|  | sS1 | 145 | | C → T | | | Leu | | 1 | | T46 | |
|  | sS2 | 1338 | | C → T | | | Gly | | 1 | | 02_1987 | |
| ***ppe46* Sublineage II (PPW subfamily)**  7 isolates  1305 bp | **Homologous recombination** | | | | | | | | | | | |
|  | HC1 | 596 - 976 | | 380 bp deletion / 383 bp insertion | | |  | | 4 | | K85, F11, CDC1551, and KZN 1435.  Results from recombination with *ppe47*. | |
|  | **In-frame insertion/ deletion** | | | | | | | | | | | |
|  | Indel1 | 4 - 7 | | 4 bp deletion / 13 bp insertion | | |  | | 1 | | *M. bovis* | |
|  | **nsSNP** | | | | | | | | | | | |
|  | nsS1 | 149 | | T → C | | | Val → Ala | | 1 | | *M. bovis* | |
|  | nsS2 | 973 | | C → G | | | Leu → Val | | 1 | | *M. bovis.* | |
| ***ppe48*/*ppe47***  **Sublineage II (PPW subfamily)**  8 isolates  1077 bp | **Homologous recombinations** | | | | | | | | | | | |
|  | HC1 | 596 - 929 | | 333 bp deletion / 330 bp insertion | | |  | | 1 | | 02_1987. Results from recombination  with *ppe46*. | |
|  | HC2 | 596 - 977 | | 381 bp deletion / 378 bp insertion | | |  | | 1 | | Haarlem. Results from recombination  with *ppe46*. | |
|  | **Frameshift** | | | | | | | | | | | |
|  | FS1 | 244 | | 1 bp deletion | | | Premature stop | | 1 | | H37Rv. New H37Rv gene (*ppe47*)  predicted to start at position 231. | |
|  | **nsSNP** | | | | | | | | | | | |
|  | nsS1 | 149 | | T → C | | | Val → Ala | | 1 | | *M. bovis* | |
| ***ppe66***  16 isolates  946 bp | **Partial Gene Deletion** | | | | | | | | | | | |
|  | PGD1 | | 1166bp deletion deletes *ppe67* and N-terminus of *ppe66*. | | | | | | 1 | | *M. bovis* | |
|  | **Frameshift** | | | | | | | | | | | |
|  | FS1 | | 897 | | 1 bp insertion | | Premature stop | | 1 | | GM1503 | |
|  | **nsSNPs** | | | | | | | | | | | |
|  | nsS1 | | 512 | | C → A | | Thr → Asn | | 4 | | EAI specific | |
|  | nsS2 | | 706 | | G → T | | Val → Leu | | 1 | | EAS054 | |
| ***ppe1***  **Sublineage II (PPW subfamily)**  18 isolates  1392 bp | **nsSNPs** | | | | | | | | | | | |
|  | nsS1 | 413 | | T → C | | | Val → Ala | | 3 | | Beijing specific | |
|  | nsS2 | 476 | | C → T | | | Thr → Met | | 1 | | Haarlem | |
|  | nsS3 | 544 | | C → T | | | Arg → Trp | | 1 | | *M. bovis* | |
|  | nsS4 | 803 | | T → C | | | Leu → Pro | | 3 | | EAI (Philippines lineage) specific | |
|  | nsS5 | 1364 | | C → T | | | Thr → Ile | | 1 | | K85 | |
|  | **sSNPs** | | | | | | | | | | | |
|  | sS1 | 894 | | G → A | | | Pro | | 1 | | K85 | |
|  | sS2 | 945 | | C → G | | | Pro | | 1 | | Strain C | |
|  | sS3 | 1011 | | C → A | | | Pro | | 2 | | K85 and *M. bovis* | |
| ***ppe20***  **Sublineage II (PPW subfamily)**  14 isolates  1620 bp | **nsSNPs** | | | | | | | | | | | |
|  | nsS1 | 171 | | G → C | | | Glu → Asp | | 1 | | T85 | |
|  | nsS2 | 281 | | T → C | | | Val → Ala | | 1 | | *M. bovis* | |
|  | nsS3 | 1327 | | G → T | | | Ala → Ser | | 1 | | 94_M4241A | |
|  | nsS4 | 1415 | | C → T | | | Pro → Leu | | 1 | | T17. Confirmed. | |
|  | nsS5 | 1445 | | C → T | | | Ala → Val | | 1 | | K85. Confirmed. | |
|  | **sSNP** | | | | | | | | | | | |
|  | sS1 | 135 | | A → C | | | Ser | | 1 | | *M. bovis* | |
| ***ppe36***  **Sublineage III**  18 isolates  732 bp | **Partial gene deletion** | | | | | | | | | | | |
|  | PGD1 | 1 – 124 | | 5’ 124 bp deletion | | |  | | 1 | | F11. Deletion is IS*6110* associated. Deletion also involves adjacent gene *pe22*. | |
|  | **Frameshift** | | | | | | | | | | | |
|  | FS1 | 136 | | 1 bp insertion | | | Premature stop | | 1 | | Strain C | |
|  | **nsSNP** | | | | | | | | | | | |
|  | nsS1 | 539 | | A → C | | | Glu → Ala | | 1 | | T92. Confirmed. | |
| ***ppe69***  **Sublineage III**  15 isolates  1200 bp | **Partial gene deletion** | | | | | | | | | | | |
|  | PGD1 | 1 - 58 | | 58 bp deletion | | | 1^st^ 23 amino acids deleted | | 1 | | Haarlem. Deletion of 5’ gene region.  Predicted alternate start codon at  position 70. | |
|  | **nsSNPs** | | | | | | | | | | | |
|  | nsS1 | 341 | | A → T | | | Glu → Val | | 1 | | CDC1551 | |
|  | nsS2 | 610 | | G → T | | | Asp → Cys | | 1 | | CDC1551 | |
|  | nsS3 | 611 | | A → G | | | Asp → Cys | | 1 | | CDC1551 | |
|  | **sSNPs** | | | | | | | | | | | |
|  | sS1 | 204 | | G → A | | | Ala | | 2 | | CDC1551 and strain C | |
|  | sS2 | 366 | | C → T | | | Asp | | 2 | | EAI isolates T46 and EAS054 | |
|  | sS3 | 609 | | T → A | | | Gly | | 1 | | CDC1551 | |
| ***ppe41***  **Sublineage III**  16 isolates  585 bp | **Partial gene deletion** | | | | | | | | | | | |
|  | PGD1 | 1 - 117 | | 117 bp deletion | | | 1st 39 amino acids deleted | | 1 | | GM 1503. Genomic deletion spans 3’  region of upstream gene (*pe25*) and 5’  region of *ppe41*. | |
|  | **sSNP** | | | | | | | | | | | |
|  | sS1 | 177 | | A → C | | | Pro | | 1 | | EAS054 | |
| ***ppe57***  **Sublineage III**  16 isolates  531 bp | **Whole gene deletions**  98-R604 INH-RIF-EM, Haarlem, strain C, KZN1435, GM1503, CDC1551. | | | | | | | | | | | |
|  | **Homologous recombination** | | | | | | | | | | | |
|  | Multiple instances of homologous recombination between the highly homologous *ppe57*, *ppe58* and *ppe59* genes. | | | | | | | | | | | |
| ***ppe58***  **Sublineage III**  16 isolates  522 bp | **Whole gene deletions**  98-R604 INH-RIF-EM, Haarlem, strain C, KZN1435, GM1503, CDC1551, *M. bovis*. | | | | | | | | | | | |
|  | **Homologous recombination** | | | | | | | | | | | |
|  | Multiple instances of homologous recombination between the highly homologous *ppe57*, *ppe58* and *ppe59* genes. | | | | | | | | | | | |
| ***ppe59***  **Sublineage III**  18 isolates  537 bp | **Whole gene deletion**  *M. bovis* | | | | | | | | | | | |
|  | **Homologous recombination** | | | | | | | | | | | |
|  | Multiple instances of homologous recombination between the highly homologous *ppe57*, *ppe58* and *ppe59* genes. | | | | | | | | | | | |
| ***ppe9***  **Sublineage IV (SVP subfamily)**  9 isolates  543 bp | **In frame deletion** | | | | | | | | | | | |
|  | D1 | 1146 - 1154 | | 9 bp deletion | | |  | | 2 | | EAI (Philippines lineage)  isolates T17 and T46 | |
|  | **Frameshifts** | | | | | | | | | | | |
|  | FS1 | 970 | | 1 bp insertion | | | Premature stop | | 1 | | T85 | |
|  | **nsSNPs** | | | | | | | | | | | |
|  | nsS1 | 425 | | A → C | | | Glu → Ala | | 1 | | *M. bovis* | |
| ***ppe17***  **Sublineage IV (SVP subfamily**  18 isolates  1041 bp | **Frameshift** | | | | | | | | | | | |
|  | FS1 | 501 | | 1 bp insertion | | | Premature stop | | 1 | | *M. bovis* (*ppe17a*) | |
|  | **nsSNP** | | | | | | | | | | | |
|  | nsS1 | 500 | | C → T | | | Pro → Leu | | 2 | | Beijing isolates 02_1987 and T85 | |
|  | **sSNP** | | | | | | | | | | | |
|  | sS1 | 675 | | C → T | | | Pro | | 1 | | K85 | |
| ***ppe29* Sublineage IV (SVP subfamily**  13 isolates  1272 bp | **Frameshifts** | | | | | | | | | | | |
|  | FS1 | 641 | | 1 bp insertion | | | No premature stop. | | 1 | | Haarlem | |
|  | **nsSNPs** | | | | | | | | | | | |
|  | nsS1 | 353 | | T → G | | | Val → Gly | | 1 | | CPHL_A | |
|  | nsS2 | 439 | | G → T | | | Ala → Ser | | 1 | | K85. Confirmed. | |
|  | nsS3 | 731 | | C → A | | | Ala → Glu | | 1 | | K85. Confirmed. | |
|  | nsS4 | 1096 | | G → C | | | Ala → Glu | | 1 | | CPHL_A | |
|  | **sSNP** | | | | | | | | | | | |
|  | sS1 | 1105 | | T → G | | | Leu | | 1 | | 94_M4241A | |
| ***ppe30***  **Sublineage IV (SVP subfamily**  15 isolates  1392 bp | **IS*6110* integration** | | | | | | | | | | | |
|  | IS1 | 1294 | |  | | |  | | 1 | | T46 | |
|  | **nsSNPs** | | | | | | | | | | | |
|  | nsS1 | 484 | | G → T | | | Gln → Stop | | 1 | | K85 | |
|  | nsS2 | 1202 | | C → T | | | Ser → Leu | | 1 | | CDC1551 | |
| ***ppe31***  **Sublineage IV (SVP subfamily**  17 isolates  1200 bp | **nsSNPs** | | | | | | | | | | | |
|  | nsS1 | 287 | | C → G | | | Ala → Gly | | 1 | | GM1503 | |
|  | nsS2 | 500 | | A → C | | | Gln → Pro | | 1 | | K85 | |
|  | nsS3 | 574 | | C → T | | | His → Tyr | | 1 | | CPHL_A | |
|  | nsS4 | 680 | | C → T | | | Ser → Phe | | 1 | | H37Rv | |
|  | nsS5 | 712 | | C → G | | | Leu → Val | | 1 | | H37Rv | |
|  | **sSNP** | | | | | | | | | | | |
|  | sS1 | 1110 | | C → T | | | Pro | | 1 | | *M. bovis* | |
| ***ppe32***  **Sublineage IV (SVP subfamily**  18 isolates  1230 bp | **nsSNP** | | | | | | | | | | | |
|  | nsS1 | 901 | | C → G | | | Ala → Gly | | 8 | | PGG2 and 3 specific | |
|  | **sSNPs** | | | | | | | | | | | |
|  | sS1 | 141 | | G → A | | | Ser | | 11 | | TBD1- specific | |
| ***ppe33***  **Sublineage IV (SVP subfamily**  17 isolates  1407 bp | **nsSNPs** | | | | | | | | | | | |
|  | nsS1 | 568 | | C → T | | | Gln → stop | | 1 | | *M. bovis*. New gene (*ppe33b*)  predicted to begin at position 571. | |
|  | nsS2 | 760 | | C → G | | | Leu → Val | | 1 | | H37Rv | |
|  | nsS3 | 1201 | | G → A | | | Gly → Arg | | 1 | | K85 | |
|  | nsS4 | 1412 | | C → G | | | Ser → stop | | 11 | | TBD1- specific. Results in loss of 2  C-terminal amino acids. | |
|  | **sSNP** | | | | | | | | | | | |
|  | sS1 | 471 | | C → T | | | Ala | | 1 | | H37Rv | |
| ***ppe65***  **Sublineage IV (SVP subfamily**  17 isolates  1242 bp | **Whole gene deletion** | | | | | | | | | | | |
|  | WGD1 |  | |  | | |  | | 2 | | K85 and *M. bovis.* Part of a 5894 bp  deletion in *M. bovis* compared to H37Rv. | |
|  | **nsSNPs** | | | | | | | | | | | |
|  | nsS1 | 83 | | C → G | | | Ala → Gly | | 1 | | Strain C | |
|  | nsS2 | 1066 | | G → A | | | Ala → Thr | | 1 | | T17. Confirmed. | |
|  | **sSNPs** | | | | | | | | | | | |
|  | sS1 | 339 | | C → T | | | Ala | | 1 | | CDC1551 | |
|  | sS2 | 381 | | T → G | | | Leu | | 1 | | CPHL_A | |
|  | sS3 | 777 | | C → A | | | Ala | | 4 | | TBD1+ specific | |
| ***ppe14***  **Sublineage IV (SVP subfamily**  18 isolates  1272 bp | **nsSNPs** | | | | | | | | | | | |
|  | nsS1 | 481 | | G → T | | | Ala → Ser | | 1 | | Haarlem | |
|  | nsS2 | 878 | | C → T | | | Thr → Met | | 1 | | CPHL_A. Confirmed. | |
|  | **sSNP** | | | | | | | | | | | |
|  | sS1 | 1200 | | C → A | | | Gly | | 1 | | 02_1987 | |
| ***ppe50***  **Sublineage IV (SVP subfamily**  18 isolates  399 bp | Hypervariable at macromutational scale [32]. Whole gene deletion in 8 isolates (CDC1551, Haarlem, strain C, 94_M4241A,  T17, T92, T46, EAS054). | | | | | | | | | | | |
| ***ppe51***  **Sublineage IV (SVP subfamily**  17 isolates  1143 bp | No variation detected. | | | | | | | | | | | |
| ***ppe61***  **Sublineage IV (SVP subfamily)**  18 isolates  1221 bp | **In-frame deletion** | | | | | | | | | | | |
|  | D1 | 82 - 84 | | 3 bp deletion | | |  | | 2 | | EAS054 and 94_M4241A.  Convergent mutation. | |
|  | **Frameshift** | | | | | | | | | | | |
|  | FS1 | 796 | | 5 bp insertion | | | Premature stop | | 1 | | CPHL_A. Confirmed. | |
|  | **nsSNPs** | | | | | | | | | | | |
|  | nsS1 | 421 | | C → T | | | Gln → stop | | 1 | | CDC1551 | |
|  | nsS2 | 770 | | C → T | | | Thr → Met | | 4 | | EAI specific | |
|  | nsS3 | 1100 | | C → T | | | Ala → Val | | 1 | | K85 | |
|  | **sSNP** | | | | | | | | | | | |
|  | sS1 | 942 | | G → C | | | Ser | | 1 | | Strain C | |
| ***ppe44***  **Sublineage IV (SVP subfamily)**  18 isolates  1149 bp | **nsSNPs** | | | | | | | | | | | |
|  | nsS1 | 176 | | G → T | | | Gly → Val | | 4 | | EAI specific | |
|  | nsS2 | 581 | | T → C | | | Phe → Ser | | 8 | | PGG1 specific. | |
|  | **sSNP** | | | | | | | | | | | |
|  | sS1 | 624 | | C → T | | | Ala | | 1 | | *M. bovis* | |
| ***ppe15***  **Sublineage IV (SVP subfamily)**  16 isolates  1176 bp | **Frameshifts** | | | | | | | | | | | |
|  | FS1 | 8 | | 1 bp deletion | | | Alternate start site at position 43 predicted. | | 1 | | 02_1987. Ppe motif absent. | |
|  | FS2 | 23 | | 1 bp deletion | | | Alternate start site at position 43 predicted. | | 1 | | 94_M4241A. Ppe motif absent. | |
|  | **nsSNPs** | | | | | | | | | | | |
|  | nsS1 | 199 | | G → A | | | Ala → Thr | | 1 | | KZN1435 | |
|  | nsS2 | 541 | | G → T | | | Ala → Ser | | 1 | | EAS054 | |
| ***ppe43***  **Sublineage IV (SVP subfamily)**  17 isolates  1185 bp | **Frameshift** | | | | | | | | | | | |
|  | FS1 | 448 - 452 | | 5 bp deletion | | | Premature stop | | 1 | | CPHL_A. Confirmed. | |
|  | **nsSNPs** | | | | | | | | | | | |
|  | nsS1 | 788 | | C → G | | | Pro → Arg | | 1 | | *M. bovis* | |
|  | nsS2 | 1040 | | G → T | | | Gly → Val | | 4 | | LAM specific | |
| ***ppe18***  **Sublineage IV (SVP subfamily)**  16 isolates  1176 bp | **Homologous recombinations** | | | | | | | | | | | |
|  | Multiple instances of homologous recombination events between the highly homologous *PPE19*, *PPE18* and *PPE60*  genes. | | | | | | | | | | | |
| ***ppe19***  **Sublineage IV (SVP subfamily)**  18 isolates  1191 bp | **Homologous recombinations** | | | | | | | | | | | |
|  | Multiple instances of homologous recombination events between the highly homologous *PPE19*, *PPE18* and *PPE60*  genes. | | | | | | | | | | | |
| ***ppe60***  **Sublineage IV (SVP subfamily)**  15 isolates  1182 bp | **Homologous recombinations** | | | | | | | | | | | |
|  | Multiple instances of homologous recombination events between the highly homologous *PPE19*, *PPE18* and *PPE60*  genes. | | | | | | | | | | | |
| ***ppe22***  **Sublineage IV (SVP subfamily)**  14 isolates  1158 bp | **nsSNPs** | | | | | | | | | | | |
|  | nsS1 | | 454 | | T → C | Tyr → His | | 1 | | | 02_1987 | |
|  | nsS2 | | 770 | | T → C | Ile → Thr | | 1 | | | 98-R604_INH-RIF-EM | |
|  | nsS3 | | 937 | | G → C | Val → Leu | | 7 | | | PGG2 and 3 specific | |
|  | nsS4 | | 1091 | | C → T | Thr → Met | | 1 | | | CDC1551 | |
| ***ppe26***  **Sublineage IV (SVP subfamily)**  17 isolates  1182 bp | **In-frame deletion** | | | | | | | | | | | |
|  | D1 | | 547 - 552 | | 6 bp deletion |  | | 1 | | | Haarlem | |
|  | **nsSNPs** | | | | | | | | | | | |
|  | nsS1 | | 241 | | G → A | Ala → Thr | | 1 | | | 02_1987 | |
|  | nsS2 | | 820 | | T → G | Ser → Ala | | 1 | | | *M. bovis* | |
|  | nsS3 | | 823 | | G → A | Ala → Thr | | 1 | | | T17. Confirmed. | |
| ***ppe23***  **Sublineage IV (SVP subfamily)**  18 isolates  1185 bp | **nsSNP** | | | | | | | | | | | |
|  | nsS1 | | 109 | | T → C | Ser → Pro | | 1 | | | K85 | |
| ***ppe45***  **Sublineage IV (SVP subfamily)**  16 isolates  1227 bp | **nsSNPs** | | | | | | | | | | | |
|  | nsS1 | | 225 | | G → A | Trp → stop | | 1 | | | K85 | |
|  | nsS2 | | 320 | | C → T | Pro → Leu | | 1 | | | 94_M4241A | |
|  | **sSNP** | | | | | | | | | | | |
|  | sS1 | | 1227 | | G → A | stop | | 1 | | | T92. Sequence error. Normal sequence confirmed. This variation not  included in analysis. | |
| ***ppe25***  **Sublineage IV (SVP subfamily)**  15 isolates  1098 bp | **Homologous recombinations** | | | | | | | | | | | |
|  | Various combinations of 10 SNPs and a 45 bp deletion | | | | | | | 4 | | | 02_1987, F11, KZN1435 and CPHL_A.  Mutations indicate recombination  with *ppe27*. | |
|  | **In-frame deletion** | | | | | | | | | | | |
|  | D1 | | 825 - 827 | | 3 bp deletion |  | | 1 | | | *M. bovis* | |
|  | **nsSNPs** | | | | | | | | | | | |
|  | nsS1 | | 164 | | C → T | Ala → Val | | 1 | | | 98-R604_INH-RIF-EM | |
|  | nsS2 | | 848 | | C → T | Ala → Val | | 3 | | | CDC1551, strain C and Haarlem | |
|  | nsS3 | | 932 | | T → G | Val → Gly | | 1 | | | *M. bovis* | |
|  | **sSNP** | | | | | | | | | | | |
|  | sS1 | | 423 | | C → T | Ala | | 1 | | | GM1503 | |
| ***ppe27***  **Sublineage IV (SVP subfamily)**  16 isolates  1053 bp | **nsSNPs** | | | | | | | | | | | |
|  | nsS1 | | 163 | | G → C | Ala → Pro | | 1 | | | K85 | |
|  | nsS2 | | 568 | | C → T | Pro → Ser | | 1 | | | *M. bovis* | |
|  | **sSNPs** | | | | | | | | | | | |
|  | sS1 | | 543 | | A → C | Ala | | 3 | | | EAI isolates EAS054, T46 and T92. | |
|  | sS2 | | 765 | | A → G | Pro | | 1 | | | *M. bovis* | |
| ***ppe38*/*ppe71***  **Sublineage IV (SVP subfamily)**  18 isolates  1176 bp | Hypervariable on a macro-mutational scale due to numerous instances of homologous recombination with identical  homologue *ppe71* plus numerous IS*6110*-associated mutations. Micro-mutations (SNPs, small indels) are  uncommon [26]. | | | | | | | | | | | |
| ***ppe49***  **Sublineage IV (SVP subfamily)**  18 isolates  1176 bp | **nsSNP** | | | | | | | | | | | |
|  | nsS1 | 547 | | C → T | | | Gln → stop | | 1 | | Haarlem | |
| ***ppe10***  **Sublineage V (MPTR subfamily)**  14 isolates  1464 bp | **Frameshifts** | | | | | | | | | | | |
|  | FS1 | | 505 | 1 bp deletion | | | Premature stop | | 1 | | T85 | |
|  | **nsSNP** | | | | | | | | | | | |
|  | nsS1 | | 23 | G → A | | | Trp → stop | | 1 | | *M. bovis*. Eighth aa coverted to stop.  Coding resumes at codon 9 resulting in  gene with 8 N-terminal aa missing. | |
|  | nsS2 | | 36 | G → T | | | Glu → Asp | | 1 | | T92. Changes Glu of ppe signature  sequence. | |
|  | nsS3 | | 863 | G → C | | | Gly → Ala | | 1 | | K85 | |
|  | nsS4 | | 1400 | C → T | | | Pro → Leu | | 1 | | K85 | |
| ***ppe12***  **Sublineage V (MPTR subfamily)**  17 isolates  1938 bp | **In-frame insertions** | | | | | | | | | | | |
|  | I1 | | 1043 | 30 bp insertion | | |  | | 3 | | EAI (Philippines lineage) specific | |
|  | **Frameshifts** | | | | | | | | | | | |
|  | FS1 | | 1125 | 1 bp deletion | | | Premature stop | | 1 | | T92. Sequence error. Normal sequence  confirmed. This variation not included in  analysis. | |
|  | **nsSNPs** | | | | | | | | | | | |
|  | nsS1 | | 1634 | A → G | | | Lys → Arg | | 11 | | TBD1- specific | |
|  | **sSNPs** | | | | | | | | | | | |
|  | sS1 | | 1389 | T → C | | | Ile | | 1 | | F11. Confirmed. | |
| ***ppe21***  **Sublineage V (MPTR subfamily)**  17 isolates  2283bp | **Frameshift** | | | | | | | | | | | |
|  | FS1 | | 60 | 1 bp deletion | | | Premature stop | | 1 | | H37Rv | |
|  | **nsSNPs** | | | | | | | | | | | |
|  | nsS1 | | 107 | C → G | | | Pro → Arg | | 1 | | *M. bovis* | |
|  | nsS2 | | 225 | G → A | | | TGG → stop | | 1 | | 94_M4241A | |
|  | nsS3 | | 449 | T → C | | | Val → Ala | | 1 | | *M. bovis* | |
|  | nsS4 | | 1844 | G → A | | | Gly → Asp | | 1 | | Haarlem | |
| ***ppe39***  **Sublineage V (MPTR subfamily)**  14 isolates  1869 bp | **IS*6110* integrations** | | | | | | | | | | | |
|  | IS1 | | 47 | |  | Premature stop | | | | 2 | Haarlem and F11. Convergent mutation [27]. | |
|  | IS2 | | 19 | |  | Premature stop | | | | 1 | H37Rv | |
|  | **Homologous recombination** | | | | | | | | | | | |
|  | HR1 | | 550 | | Fusion with *PPE40* |  | | | | 2 | K85 and 94_M4241A. Convergent  mutation. | |
|  | **Whole gene deletions** | | | | | | | | | | | |
|  | WGD1 | |  | |  |  | | | | 1 | T92. Part of large RD5-like deletion. | |
|  | WGD2 | |  | |  |  | | | | 1 | 02_1987. Part of a major genomic  rearrangement [26]. | |
|  | **Partial gene deletion** | | | | | | | | | | | |
|  | PGD1 | | 1358 | |  | Premature stop | | | | 1 | *M. bovis.* *Ppe39* part of the RD5  deletion. | |
|  | **In-frame deletions** | | | | | | | | | | | |
|  | D1 | | 88 - 90 | | 3 bp deletion |  | | | | 2 | EAS054 and CDC1551.  Convergent mutation. | |
|  | **nsSNP** | |  | |  |  | | | |  |  | |
|  | nsS1 | | 539 | | T → C | Leu → Ser | | | | 1 | 98-R604_INH-RIF-EM | |
| ***ppe40***  **Sublineage V (MPTR subfamily)**  16 isolates  1848bp | **IS*6110* integrations** | | | | | | | | | | | |
|  | IS1 | | 47 | |  | Premature stop | | | | 2 | 02_1987 and CPHL_A. Convergent  mutation [27]. | |
|  | **Homologous recombination** | | | | | | | | | | | |
|  | HR1 | | 550 | | Fusion with *ppe39* |  | | | | 2 | K85 and 94_M4241A. Convergent  mutation [26]. | |
|  | **In-frame deletions** | | | | | | | | | | | |
|  | D1 | | 490 - 492 | | 3 bp deletion |  | | | | 1 | *M. bovis* | |
|  | **Partial gene deletion** | | | | | | | | | | | |
|  | PGD1 | | 1582 | | RD5-like deletion | Premature stop | | | | 1 | T92. Large deletion fuses 5’ region of  *PPE40* with *plcC*. | |
|  | **nsSNP** | | | | | | | | | | | |
|  | nsS1 | | 1096 | | G → C | Gly → Arg | | | | 1 | KZN 1435 | |
|  | nsS2 – S6 | | 1100 - 1004 | | CTGGA →  ACAAC | Thr, Gly →  Asn, Asn | | | | 1 | KZN 1435. nsS1-6 represents 6 SNPs  in a 9 bp region. | |
|  | **sSNP** | | | | | | | | | | | |
|  | sS1 | | 969 | | T → C | Asn | | | | 1 | Strain C | |
| ***ppe6***  **Sublineage V (MPTR subfamily)**  15 isolates  2892 bp  This gene split into 2 (*ppe5/6*) in bovis, K85 (type 1) and H37Rv, T17 (type 2). This gene split into 3 predicted open reading frames in CPHL_A. | **In-frame insertions** | | | | | | | | | | | |
|  | I1 | | 2379 | | 30 bp insertion |  | | | | 1 | KZN 1435 | |
|  | I2 | | 7604 | | 15 bp insertion |  | | | | 5 | PGG2 and 3 specific | |
|  | **In-frame deletions** | | | | | | | | | | | |
|  | D1 | | 834 - 842 | | 9 bp deletion |  | | | | 1 | 94_M4241A | |
|  | D2 | | 4763 - 4822 | | 60 bp deletion |  | | | | 1 | F11. Confirmed. | |
|  | **Frameshifts** | |  | |  |  | | | |  |  | |
|  | FS1 | | 2399 | | 1 bp insertion | Premature stop | | | | 1 | H37Rv  CPHL_A. FS1 and 2 both result in a  new gene (*ppe5*) starting at *ppe6*  codon 983. | |
|  | FS2 | | 2930 | | 1 bp deletion | Premature stop | | | | 1 |  |  |
|  | FS3 | | 5945 | | 1 bp deletion | Premature stop | | | | 2 | CPHL_A, K85 and *M. bovis* specific.  Results in new gene (*ppe5* starting at  *ppe6* codon 2035. Note alternate *ppe5* to that formed from FS1-3. Third *ppe*  gene formed in CPHL_A (see *ppe*  FS1). | |
|  | **nsSNP** | | | | | | | | | | | |
|  | nsS1 | | 617 | | G → C | Gly → Ala | | | | 3 | EAI (Philippines lineage) specific | |
|  | nsS2 | | 2727 | | A → G | Ile → Met | | | | 1 | 94_M4241A | |
|  | nsS3 | | 2798 | | G → T | Gly → Val | | | | 4 | LAM and PGG3 specific | |
|  | nsS4 | | 6118 | | G → A | Asp → Asn | | | | 1 | 02_1987 | |
|  | nsS5 | | 6763 | | A → G | Ile → Val | | | | 3 | EAI specific. Same mutation seen in  *ppe5* nsS1. | |
|  | nsS6 | | 7358 | | G → A | Gly → Glu | | | | 2 | EAI (Philippine lineage) specific. Same  mutation seen in *ppe5* nsS2. | |
|  | nsS7 | | 8219 | | G → C | Gly → Ala | | | | 1 | 94_M4241A | |
|  | nsS8 | | 8240 | | G → C | Phe → Ser | | | | 8 | TBD1- specific. Same mutation as seen  in *ppe5* nsS4. | |
|  | nsS9 | | 9412 | | A → G | Asn → Asp | | | | 1 | T92. Confirmed. | |
|  | nsS10 | | 9464 | | G → A | Gly → Asp | | | | 1 | T92. Confirmed. | |
|  | **sSNP** | | | | | | | | | | | |
|  | sS1 | | 1359 | | T → C | Arg | | | | 1 | T85 | |
|  | sS2 | | 2928 | | G → A | Gly | | | | 1 | Haarlem | |
|  | sS3 | | 3562 | | T → C | Leu | | | | 8 | TBD1- specific | |
|  | sS4 | | 4446 | | G → A | Ser | | | | 1 | F11. Confirmed. | |
|  | sS5 | | 5135 | | G → A | Pro | | | | 1 | T46 | |
|  | sS6 | | 5454 | | C → G | Thr | | | | 1 | 02_1987 | |
|  | sS7 | | 5655 | | C → T | Gly | | | | 1 | EAS054 | |
| ***ppe5***  **Sublineage V (MPTR subfamily)**  15 isolates  6615 bp  *Ppe5* formed from split of *ppe6*. Only present in *M. bovis*, K85 & CPHL_A (type 1 *PPE5*) and Rv, T17 and CPHL_A (type 2).  CPHL_A *ppe5* further split into additional gene. | **In-frame insertions** | | | | | | | | | | | |
|  | I1 | | 4658 | | 15 bp insertion |  | | | | 1 | H37Rv. Same insertion as seen in  ppe6 I2. | |
|  | **Frameshifts** | | | | | | | | | | | |
|  | FS1 | | 2929 | | 1 bp deletion | Premature stop | | | | 1 | CPHL_A. Results in 3^rd^ *ppe* gene  Starting at *ppe6* codon 2035 (see  *ppe6* FS4). Confirmed. | |
|  | **nsSNP** | |  | |  |  | | | |  |  | |
|  | nsS1 | | 1441 | | G → A | Gly → Thr | | | | 2 | *M. bovis* & K85 specific | |
|  | nsS2 | | 5295 | | G → C | Phe → Ser | | | | 1 | H37Rv. Same mutation seen in *ppe6*  nsS8. | |
|  | nsS3 | | 2863 | | A → G | Thr → Ala | | | | 1 | *M. bovis* | |
|  | **sSNP** | | | | | | | | | | | |
|  | sS1 | | 616 | | T → C | Leu | | | | 1 | H37Rv (same mutation seen in  *ppe6* sS3). | |
|  | sS2 | | 1212 | | T → C | Gly | | | | 1 | K85 | |
| ***ppe54***  **Sublineage V (MPTR subfamily)**  10 isolates  7572 bp | Extreme variation observed. All isolates unique.  Note: Identical gene sequence in the closely related isolates KZN1435, 4207 & 605. Identical gene sequence in 3 members  of the Harlingen transmission chain [58,59 (average coverage = 84%). | | | | | | | | | | | |
| ***ppe8***  **Sublineage V (MPTR subfamily)**  12 isolates  9903 bp    1 gene (*ppe8*) in TBD1+. Two genes (*ppe7* & *ppe8*) in TBD1- due to frameshift (FS2) with termination in *ppe8* and new start site. | **In-frame deletions** | | | | | | | | | | | |
|  | D1 | | 6434 - 6493 | 60 bp deletion | | |  | | | 2 | LCC (CDC1551 and strain C) specific | |
|  | D2 | | 7506 - 7535 | 30 bp deletion | | |  | | | 1 | Strain C | |
|  | D3 | | 9311 - 9370 | 60 bp deletion | | |  | | | 3 | EAS054, KZN 1435 and 98-R604_INH-RIF-EM. Possible convergence. | |
|  | **In-frame insertions** | | | | | | | | | | | |
|  | I1 | | 5003 | 15 bp insertion | | |  | | | 1 | 98-R604_INH-RIF-EM | |
|  | I2 | | 7352 | 15 bp insertion | | |  | | | 1 | H37Rv | |
|  | **Frameshifts** | | | | | | | | | | | |
|  | FS1 | | 8947 | 1 bp insertion | | | Premature stop | | | 1 | Strain C | |
|  | FS2 | | 9875 | 2 bp deletion | | | Premature stop | | | 8 | All TBD1+ isolates. Coding region for  *ppe7* begins at position 9973 for TBD1+  isolates. | |
|  | **nsSNPs** | | | | | | | | | | | |
|  | nsS1 | | 353 | T → C | | | Val → Ala | | 2 | | EAI specific | |
|  | nsS2 | | 1240 | T → G | | | Phe → Val | | 1 | | CPHL_A. Confirmed. | |
|  | nsS3 | | 3578 | C → G | | | Ala → Gly | | 1 | | EAS054 | |
|  | nsS4 | | 4027 | G → A | | | Gly → Ser | | 1 | | K85 | |
|  | nsS5 | | 4639 | G → T | | | Ala → Ser | | 1 | | K85 | |
|  | nsS6 | | 5520 | A → G | | | Asn → Asp | | 1 | | *M. bovis* | |
|  | nsS7 | | 5840 | G → A | | | Gly → Asp | | 1 | | Haarlem | |
|  | nsS8 | | 6296 | G → A | | | Gly → Asp | | 1 | | T46 | |
|  | nsS9 | | 6337 | T → C | | | Trp → Arg | | 1 | | Strain C | |
|  | nsS10 | | 7173 | T → G | | | Ser → Arg | | 1 | | Strain C | |
|  | nsS11 | | 7756 | G → T | | | Gly → Trp | | 1 | | H37Rv | |
|  | nsS12 | | 7897 | G → A | | | Gly → Ser | | 1 | | EAI specific | |
|  | nsS13 | | 8484 | C → A | | | Phe → Leu | | 1 | | K85. Confirmed. | |
|  | nsS14 | | 9733 | T → A | | | Phe → Ile | | 8 | | All TBD1- isolates | |
|  | nsS15 | | 9931 | G → A | | | Ser → Thr | | 1 | | K85 | |
|  | nsS16 | | 10418 | T → C | | | Phe → Val | | 1 | | EAS054 | |
|  | **sSNPs** | | | | | | | | | | | |
|  | sS1 | | 657 | G → A | | | Ser | | 1 | | | 94_M4241A |
|  | sS2 | | 3357 | C → A | | | Gly | | 1 | | | K85 |
|  | sS3 | | 3924 | C → T | | | Ile | | 1 | | | Haarlem |
|  | sS4 | | 4122 | C → T | | | Asn | | 1 | | | EAS054 |
|  | sS5 | | 5433 | G → A | | | Gly | | 1 | | | F11. Confirmed. |
|  | sS6 | | 5982 | A → G | | | Ala | | 7 | | | All PGG2 and 3 isolates |
|  | sS7 | | 7209 | A → C | | | Gly | | 1 | | | *M. bovis* |
|  | sS8 | | 9684 | C → T | | | Pro | | 1 | | | 94_M4241A |
| ***ppe7***  **Sublineage V (MPTR subfamily)**  8 isolates  426 bp | **Frameshift** | | | | | | | | | | | |
|  | FS1 | | 375 | 1 bp deletion | | | Premature stop | | 1 | | | H37Rv |
|  | **nsSNPs** | | | | | | | | | | | |
|  | nsS1 | | 271 | G → T | | | Ala → Ser | | 1 | | | CDC1551 |
| ***ppe16***  **Sublineage V (MPTR subfamily)**  16 isolates  1857 bp | **Whole gene deletion** | | | | | | | | | | | |
|  | WGD1 | |  |  | | |  | | 1 | | | CPHL_A. *Ppe16* deleted along with  neighbouring gene Rv*1134*. |
|  | **IS*6110* integration** | | | | | | | | | | | |
|  | IS1 | | 1222 |  | | | Premature stop | | 1 | | | T85 |
|  | **Frameshifts** | | | | | | | | | | | |
|  | FS1 | | 1333 - 1337 | 5 bp deletion | | | Premature stop | | 1 | | | K85 |
|  | **nsSNPs** | | | | | | | | | | | |
|  | nsS1 | | 85 | T → A | | | Val → Asp | | 1 | | | T17. Confirmed. |
|  | nsS2 | | 314 | C → T | | | Ala → Val | | 1 | | | CDC1551 |
|  | **sSNP** | | | | | | | | | | | |
|  | sS1 | | 342 | G → A | | | Val | | 1 | | | K85. Confirmed. |
| ***ppe24***  **Sublineage V (MPTR subfamily)**  13 isolates  3162 bp | Extreme variation observed. All isolates unique.  Note: Identical gene sequence in the closely related isolates KZN1435, 4207 & 605. Identical gene sequence in 3 members  of the Harlingen transmission chain [58,59] (average coverage = 84%). | | | | | | | | | | | |
| ***ppe13***  **Sublineage V (MPTR subfamily)**  17 isolates  1332 bp | **Frameshifts** | | | | | | | | | | | |
|  | FS1 | | 51 | 1 bp deletion | | | Premature stop | | 1 | | | KZN 1435 |
|  | FS2 | | 1306 | 1 bp deletion | | | Poly C/poly A region from position 1298 results in numerous FS variations. | | 5 | | | PGG1and 2 isolates GM1503, T17, T46, T92 and K85. |
|  | FS3 | | 1307 | 1 bp insertion | | |  |  | 4 | | | PGG1 and 2 isolates F11, KZN 1435,  CPHL_A and EAS054. Note:  sequence reanalysis shows CPHL_A  has a 2 bp insertion. |
|  | FS4 | | 1313 | 2 bp deletion | | |  |  | 1 | | | CPHL_A |
|  | FS5 | | 1314 | 1 bp deletion | | |  |  | 9 | | | PGG1 and 2 isolates *M. bovis*, strain C, 98-R604, T17, T46, T92, K85, EAS054, GM1503. |
|  | **nsSNPs** | | | | | | | | | | | |
|  | nsS1 | | 289 | G → A | | | Ala → Ser | | 3 | | | EAI (Philippines lineage) specific |
|  | nsS2 | | 513 | G → A | | | Trp → stop | | 1 | | | EAS054 |
|  | **sSNPs** | | | | | | | | | | | |
|  | sS1 | | 732 | C → T | | | Asn | | 1 | | | K85 |
|  | sS2 | | 1008 | C → T | | | Gly | | 4 | | | EAI specific |
| ***ppe34***  **Sublineage V (MPTR subfamily)**  17 isolates  4380 bp | Extreme variation observed. All isolates unique.  Note: Identical gene sequence in the closely related isolates KZN1435, 4207 & 605. Identical gene sequence in 3 members  of the Harlingen transmission chain [58,59 (average coverage = 84%). | | | | | | | | | | | |
| ***ppe35***  **Sublineage V (MPTR subfamily)**  18 isolates  2964 bp | **In frame deletion** | | | | | | | | | | | |
|  | D1 | | 1603 - 1680 | 78 bp deletion | | |  | | 1 | | | Haarlem |
|  | **Frameshift** | | | | | | | | | | | |
|  | FS1 | | 1877 | 1 bp insertion | | | Premature stop | | 1 | | | *M. bovis*. Results in stop codon at  nucleotide position 1953 – 1955 and  new predicted gene (*ppe35b*) start  codon at position 2038. |
|  | **nsSNPs** | | | | | | | | | | | |
|  | nsS1 | | 1949 | G → A | | | Gly → Asp | | 1 | | | Strain C |
|  | nsS2 | | 1960 | C → A | | | Ser → Thr | | 1 | | | K85. Confirmed. |
|  | nsS3 | | 335 | C → T | | | Thr → Ile | | 1 | | | *M. bovis*. *Ppe35b* (see FS1). |
|  | nsS4 | | 643 | A → C | | | Ser → Arg | | 1 | | | *M. bovis*. *Ppe35b* (see FS1). |
|  | nsS5 | | 2708 | G → A | | | Gly → Asp | | 1 | | | EAS054 |
|  | nsS6 | | 2765 | C → T | | | Ser → Leu | | 8 | | | PGG2 and 3 specific |
|  | **sSNP** | | | | | | | | | | | |
|  | sS1 | | 2238 | C → G | | | Val | | 1 | | | Strain C |
| ***ppe28***  **Sublineage V (MPTR subfamily)**  17 isolates  1968 bp | **Frameshift** | | | | | | | | | | | |
|  | FS1 | | 169 - 213 | 45 bp deletion/  44 bp insertion | | | Premature stop | | 1 | | | T17. Sequence error. Normal sequence confirmed. This variation not included in analysis. |
|  | **nsSNPs** | | | | | | | | | | | |
|  | nsS1 | | 432 | G → T | | | Trp → Cys | | 10 | | | TBD1- specific |
|  | nsS2 | | 449 | C → T | | | Ala → Val | | 2 | | | Haarlem and strain C. |
|  | nsS3 | | 757 | T → G | | | Phe → Val | | 2 | | | *M. bovis* and K85 |
|  | nsS4 | | 1508 | T → C | | | Val → Ala | | 1 | | | *M. bovis* |
|  | **sSNP** | | | | | | | | | | | |
|  | sS1 | | 1509 | C → T | | | Val | | 1 | | | T46 |
| ***ppe63***  **Sublineage V (MPTR subfamily)**  15 isolates  1440 bp | **nsSNPs** | | | | | | | | | | | |
|  | nsS1 | | 1093 | T → A | | | Tyr → Asn | | 4 | | | EAI specific |
|  | nsS2 | | 1265 | C → T | | | Thr → Met | | 1 | | | K85 |
| ***ppe42***  **Sublineage V (MPTR subfamily)**  14 isolates  1743 bp | **nsSNPs** | | | | | | | | | | | |
|  | nsS1 | | 157 | G → A | | | Ala → Thr | | 1 | | | CDC1551 |
|  | nsS2 | | 841 | C → T | | | Pro → Ser | | 2 | | | *M. bovis* and K85 |
| ***ppe53***  **Sublineage V (MPTR subfamily)**  12 isolates  1773 bp | **In frame deletion** | | | | | | | | | | | |
|  | D1 | | 190 - 192 | 3 bp deletion | | |  | | 4 | | | *M. bovis*, T17, T46 and K85 |
|  | D2 | | 1186 - 1215 | 30 bp deletion | | |  | | 1 | | | EAS054 |
|  | **Frameshift** | | | | | | | | | | | |
|  | FS1 | | 60 | 1 bp deletion | | | Premature stop | | 2 | | | T17 and T46 |
|  | **nsSNPs** | | | | | | | | | | | |
|  | nsS1 | | 97 | C → G | | | Arg → Gly | | 2 | | | EAS054 and Haarlem. Possible  convergent mutation. |
|  | nsS2 | | 612 | C → A | | | Asp → Glu | | 1 | | | *M. bovis* |
|  | nsS3 | | 1013 | G → T | | | Gly → Val | | 1 | | | EAS054 |
|  | nsS4 | | 1681 | A → G | | | Thr → Ala | | 1 | | | EAS054 |
| ***ppe*62**  **Sublineage V (MPTR subfamily)**  15 isolates  1749 bp | **In frame deletion** | | | | | | | | | | | |
|  | D1 | | 207 - 209 | 3 bp deletion | | |  | | 1 | | | GM1503 |
|  | **In frame insertion** | | | | | | | | | | | |
|  | I1 | | 956 | 90 bp insertion | | |  | | 1 | | | CPHL_A |
|  | **nsSNPs** | |  |  | | |  | |  | | |  |
|  | nsS1 | | 1026 | C → A | | | Ser → Arg | | 1 | | | Haarlem |
|  | nsS2 | | 1690 | G → A | | | Gly → Ser | | 2 | | | T17 and T46 |
| ***ppe52***  **Sublineage V (MPTR subfamily)**  11 isolates  1230 bp | **Frameshift** | | | | | | | | | | | |
|  | FS1 | | 284 | 1 bp insertion | | | Premature stop | | 1 | | | T85 |
|  | **nsSNPs** | |  |  | | |  | |  | | |  |
|  | nsS1 | | 1198 | C → A | | | Gln → Lys | | 5 | | | PGG2 and 3 specific |
| ***ppe64***  **Sublineage V (MPTR subfamily)**  16 isolates  1659 bp | **In frame deletion** | | | | | | | | | | | |
|  | D1 | | 88 - 90 | 3 bp deletion | | |  | | 4 | | | LAM specific |
|  | D2 | | 590 - 649 | 60 bp deletion. | | |  | | 1 | | | K85 |
|  | **In frame insertion** | | | | | | | | | | | |
|  | I1 | | 911 | 30 bp insertion. | | |  | | 1 | | | 02_1987 |
|  | **Frameshift** | | | | | | | | | | | |
|  | FS1 | | 757 | 1 bp insertion. | | | Premature stop. | | 1 | | | T92. Confirmed. |
|  | **nsSNPs** | | | | | | | | | | | |
|  | nsS1 | | 34 | A → G | | | Asn → Asp | | 1 | | | CPHL_A |
|  | nsS2 | | 916 | G → A | | | Gly → Ser | | 4 | | | EAI specific |
|  | nsS3 | | 1019 | A → G | | | Ile → Ser | | 1 | | | GM1503 |
| ***ppe55***  **Sublineage V (MPTR subfamily)**  9 isolates  9474 bp | Extreme variation observed. Numerous frameshifts split the gene into 2 or 3 distinct open reading frames in several  isolates.  Note: Identical gene sequence in the closely related isolates KZN1435, 4207 & 605. Identical gene sequence observed in 3 members of the Harlingen transmission chain [58,59] (average coverage = 84%). | | | | | | | | | | | |
| ***ppe56***  **Sublineage V (MPTR subfamily)**  6 isolates  11151 bp | **Frameshift** | | | | | | | | | | | |
|  | FS1 | | 6476 - 6546 | 71 bp deletion and 58 bp insertion. | | | Premature stop. | | 1 | | | Haarlem |
|  | FS2 | | 6041 | 1 bp deletion | | | Premature stop. | | 1 | | | *M. bovis* (*ppe56b*). New gene (*ppe56d*) begins position 7705. Note: no *ppe56c* listed in BoviList [76]. |
|  | **nsSNPs** | | | | | | | | | | | |
|  | nsS1 | | 52 | T → C | | | Cys → Arg | | 1 | | | *M. bovis* |
|  | nsS2 | | 1518 | C → A | | | Tyr → stop | | 1 | | | *M. bovis*. New gene (*ppe56b*) begins at position 1576. |
|  | nsS3 | | 2897 | G → A | | | Gly → Asp | | 1 | | | EAS054 |
|  | nsS4 | | 1360 | A → G | | | Asn → Asp | | 1 | | | *M. bovis* (*ppe56b*) |
|  | nsS5 | | 3149 | C → T | | | Thr → Ile | | 1 | | | EAS054 |
|  | nsS6 | | 3150 | C → T | | | Thr → Ile | | 1 | | | EAS054 |
|  | nsS7 | | 5143 | G → T | | | Gly → Cys | | 1 | | | EAS054 |
|  | nsS8 | | 3878 | C → T | | | Ala → Val | | 1 | | | *M. bovis* (*ppe56b*) |
|  | **sSNPs** | | | | | | | | | | | |
|  | sS1 | | 906 | G → C | | | Gly | | 1 | | | *M. bovis* |
|  | sS2 | | 126 | C → G | | | Ala | | 1 | | | *M. bovis* (*ppe56b*) |
|  | sS3 | | 3777 | G → A | | | Glu | | 2 | | | EAI isolates EAS054 and T46 |
|  | sS4 | | 1566 | G → A | | | Ser | | 1 | | | *M. bovis* (*ppe56d*) |

Table S1B

| **Gene** | **Sequence variation** | **Position** | **Genetic change** | **Amino acid change** | **Number of isolates** | **Comments** |
| --- | --- | --- | --- | --- | --- | --- |
| ***pe35***  **Sublineage I**  17 isolates  297 bp | **Frameshift** | | | | | |
|  | FS1 | 10 | 1 bp deletion | Premature stop | 2 | 94_M4241A and CPHL_A.  Convergent mutation. Occurs in polyA sequence. |
|  | **nsSNP** | | | | | |
|  | nsS1 | 295 | T → G | Stop → Glu | 1 | H37Rv. 1 additional amino acid added to C-terminal end. |
| ***pe34***  **Sublineage I**  18 isolates  336 bp | **nsSNP** | | | | | |
|  | nsS1 | 155 | C → T | Thr → Met | 1 | GM1503 |
| ***pe5***  **Sublineage II**  18 isolates  309 bp | No variation detected. | | | | | |
| ***pe15***  **Sublineage II**  17 isolates  309 bp | **nsSNP** | | | | | |
|  | nsS1 | 151 | G → A | Ala → Thr | 1 | Haarlem |
| ***pe29***  **Sublineage II**  9 isolates  315 bp | No variation detected. | | | | | |
| **pe36**  **Sublineage III**  18 isolates  234 bp | No variation detected. | | | | | |
| ***pe25***  **Sublineage III**  18 isolates  300 bp | No variation detected. | | | | | |
| **pe22**  **Sublineage III** 18 isolates  297 bp | **Whole gene deletion** | | | | | |
|  | WGD1 |  |  |  | 1 | F11. Deletion associated with IS*6110*. Deletion also includes part of adjacent  *ppe36* gene. |
| ***pe11***  **Sublineage IV**  18 isolates  303 bp | **sSNP** | | | | | |
|  | sS1 | 90 | C → T | Asp | 1 | T85 |
| ***pe20***  **Sublineage IV**  16 isolates  300 bp | No variation detected. | | | | | |
| ***pe18***  **Sublineage IV**  18 isolates  300 bp | **IS*6110* integration** | | | | | |
|  | IS1 | 195 |  |  | 1 | 98-R604 INH-RIF-EM |
|  | **nsSNP** | | | | | |
|  | nsS1 | 4 | T → C | Ser → Pro | 1 | Haarlem |
|  | **sSNP** | | | | | |
|  | sS1 | 300 | G → A | Stop | 1 | *M. bovis* |
| ***pe19***  **Sublineage IV**  18 isolates  300 bp | **sSNP** | | | | | |
|  | sS1 | 123 | C → T | Pro | 1 | *M. bovis* |
| ***pe32***  **Sublineage IV**  18 isolates  300 bp | **Whole gene deletion** | | | | | |
|  | WGD1 |  |  |  | 2 | K85 and *M. bovis* (RD8 deletion). |
|  | **sSNP** | | | | | |
|  | sS1 | 84 | A → G | Gly | 1 | Strain C |
| ***pe13***  **Sublineage IV**  17 isolates  300 bp | No variation detected. | | | | | |
| ***pe31***  **Sublineage IV**  17 isolates  297 bp | **nsSNP** | | | | | |
|  | nsS1 | 77 | C → T | Ala → Val | 1 | *M. bovis* |
|  | **sSNP** | | | | | |
|  | sS1 | 84 | T → C | Asn | 1 | F11. Confirmed. |
| ***pe7***  **Sublineage IV**  17 isolates  300 bp | No variation detected. | | | | | |
| ***pe8***  **Sublineage IV**  16 isolates  828 bp | **nsSNP** | | | | | |
|  | nsS1 | 511 | G → A | Ala → Thr | 1 | K85. Confirmed. |
|  | **sSNPs** | | | | | |
|  | sS1 | 243 | G → A | Gly | 1 | H37Rv |
|  | sS2 | 810 | C → T | Pro | 1 | K85. Confirmed. |
| ***pe27***  **Sublineage IV**  15 isolates  828 bp | **nsSNPs** | | | | | |
|  | nsS1 | 128 | C → T | Ala → Val | 2 | EAI (Philippines lineage) isolates T17 and T46. |
|  | nsS2 | 152 | T → G | Leu → Arg | 1 | CDC1551 |
|  | nsS3 | 136 | C → T | Pro → Ser | 6 | TBD1+ isolate specific. |
|  | nsS4 | 808 | A → G | Met → Val | 5 | H37Rv, F11, 98-R604_INH-RIF-EM, GM1503 and KZN1435. |
| ***pe2***  **Sublineage V (PGRS subfamily)**  18 isolates  1578 bp | **Frameshifts** | | | | | |
|  | FS1 | 998 | 1 bp deletion | Combine to produce premature stop. | 1 | EAS054 |
|  | FS2 | 1021 | 1 bp deletion |  |  |  |
|  | FS3 | 1024 | 1 bp deletion |  |  |  |
|  | FS4 | 1027 | 1 bp deletion |  |  |  |
|  | **nsSNPs** | | | | | |
|  | nsS1 | 872 | G → A | Gly → Glu | 4 | LAM specific. |
|  | **sSNP** | | | | | |
|  | sS1 | 903 | G → A | Glu | 1 | Strain C |
| ***pe24***  **Sublineage V (PGRS subfamily)**  18 isolates  1005 bp | **nsSNP** | | | | | |
|  | nsS1 | 932 | G → A | Gly → Val | 2 | Beijing isolates 02_1987 and T85. |
| ***pe26***  **Sublineage V (PGRS subfamily)**  8 isolates  1479 bp | **nsSNPs** | | | | | |
|  | nsS1 | 518 | G → C | Gly → Ala | 1 | *M. bovis* |
|  | nsS2 | 519 | G → C | Gly → Ala | 1 | *M. bovis* |
| ***pe4***  **Sublineage V (PGRS subfamily)**  18 isolates  1509 bp | **Frameshift** | | | | | |
|  | FS1 | 360 | 1 bp deletion | Premature stop | 1 | GM1503 |
|  | **nsSNPs** | | | | | |
|  | nsS1 | 346 | G → A | Ala → Thr | 1 | *M. bovis* |
|  | nsS2 | 492 | G → C | Lys → Asn | 4 | EAI specific. |
|  | nsS3 | 631 | G → A | Ala → Thr | 2 | *M. bovis* and K85. |
|  | nsS4 | 1108 | C → T | Gln → stop | 1 | K85. Confirmed. |
|  | nsS5 | 1426 | C → G | Gln → Glu | 1 | T46 |
|  | **sSNPs** | | | | | |
|  | sS1 | 232 | C → A | Arg | 1 | T46 |
| ***pe3***  **Sublineage V (PGRS subfamily)**  17 isolates  1407 bp | **nsSNPs** | | | | | |
|  | nsS1 | 40 | G → A | Ala → Thr | 1 | H37Rv |
|  | nsS2 | 271 | G → C | Glu → Gln | 1 | 94_M4241A |
|  | nsS3 | 763 | C → A | Pro → Thr | 1 | *M. bovis* |
|  | nsS4 | 1102 | C → T | Arg → Trp | 1 | CDC1551 |
|  | **sSNP** | | | | | |
|  | sS1 | 39 | G → C | Thr | 1 | CPHL_A. Confirmed. |
| ***pe12***  **Sublineage V (PGRS subfamily)**  18 isolates  927 bp | **Frameshift** | | | | | |
|  | FS1 | 98 | 1 bp insertion | Premature stop | 1 | 02_1987 |
|  | **nsSNPs** | | | | | |
|  | nsS1 | 339 | A → C | Gln → His | 2 | *M.bovis* and K85. |
|  | nsS2 | 649 | C → T | Leu → Phe | 1 | CPHL_A. Confirmed. |
| ***pe14***  **Sublineage V (PGRS subfamily)**  15 isolates  333 bp | **Frameshift** | | | | | |
|  | FS1 | 204 - 208 | 5 bp deletion | Extended protein | 1 | *M. bovis* |
| ***pe16***  **Sublineage V (PGRS subfamily)**  15 isolates  1587 bp | **sSNP** | | | | | |
|  | sS1 |  |  |  | 2 | Beijing isolates 94_M4241A and 02_1987. |
| ***pe23***  **Sublineage V (PGRS subfamily)**  17 isolates  1149 bp | **nsSNPs** | | | | | |
|  | nsS1 | 846 | T → C | Ser → Arg | 3 | Beijing specific |
|  | nsS2 | 983 | A → G | Gln → Arg | 1 | KZN1435 |
|  | nsS3 | 1030 | G → A | Ala → Thr | 2 | Beijing isolates 02_1987 and T85. |
| ***pe17***  **Sublineage V (PGRS subfamily)**  16 isolates  933 bp | **In frame deletion** | | | | | |
|  | D1 | 464 - 466 | 3 bp deletion |  | 1 | 94_M4241A |
| ***pe1***  **Sublineage V (PGRS subfamily)**  18 isolates  1767 bp | **Frameshift** | | | | | |
|  | FS1 | 1192 - 1255 | 64 bp deletion/ 59 bp insertion | Premature stop | 1 | 98-R604. Inserted sequence derives from Rv*0446c*. |
|  | **nsSNPs** | | | | | |
|  | nsS1 | 76 | G → A | Gly → Arg | 1 | *M. bovis* |
|  | nsS2 | 364 | G → A | Ala → Thr | 3 | Haarlem, strain C and CDC1551. |
|  | nsS3 | 369 | C → A | Asn → Lys | 1 | KZN1435 |
|  | nsS4 | 1481 | C → T | Pro → Leu | 1 | K85 |
|  | nsS5 | 1714 | A → G | Ile → Val | 3 | Haarlem, strain C and CDC1551. |
|  | **sSNP** | | | | | |
|  | sS1 | 1453 | T → C | Leu | 1 | H37Rv |
| ***pe9***  **Sublineage V (PGRS subfamily)**  18 isolates  435 bp | No variation detected. | | | | | |
| ***Ppe10***  **Sublineage V (PGRS subfamily)**  15 isolates  363 bp | **Frameshift** | | | | | |
|  | FS1 | 338 | 1 bp deletion | Extended protein | 2 | Beijing isolates 02_1987 and 94_M4241A. |
|  | **nsSNP** | | | | | |
|  | nsS1 | 281 | G → A | Gly → Asp | 2 | EAI (Philippines lineage)  isolates T17 and T92. |
| ***pe33***  **Sublineage V (PGRS subfamily)**  17 isolates  285 bp | No variation detected | | | | | |
| ***pe6***  **Sublineage V (PGRS subfamily)**  14 isolates  516 bp | **Frameshift** | | | | | |
|  | FS1 | 141 | 1 bp deletion | Premature stop | 2 | EAI isolates T17 and EAS054. |
|  | **nsSNP** | | | | | |
|  | nsS1 | 470 | C → T | Leu → Phe | 1 | K85 |
