# Supplementary material for: Comparative Analysis of Mycobacterium tuberculosis pe and ppe Genes Reveals High Sequence Variation and an Apparent Absence of Selective Constraints
Source: PLoS One. 2012 Apr 4;7(4):e30593. doi: 10.1371/journal.pone.0030593 (PMC3319526; doi:10.1371/journal.pone.0030593)
Supplement: Table S4 — (DOCX) [file pone.0030593.s004.docx]

**Table S4.**

| **Isolate** | **Gene** | **Variation** | **Result** |
| --- | --- | --- | --- |
| F11 | *ppe11* | nsS2. Position 1510. A → G | Confirmed |
| F11 | *ppe 12* | sS1. Position 1389. T → C | Confirmed |
| F11 | *ppe 5/6* | sS4. Position 4446. G → A | Confirmed |
| F11 | *ppe 5/6* | D2. Position 4763 – 4822. 60 bp deletion | Confirmed |
| F11 | *ppe7/8* | sS5. Position 5433. G → A | Confirmed |
| F11 | *ppe 13* | FS4/5. Poly C/A region | Confirmed |
| F11 | *pe31* | sS1. Position 84. T → C | Confirmed |
| CPHL_A | *ppe5/6* | FS1. Position 2929. 1 bp deletion | Confirmed |
| CPHL_A | *ppe37* | nsS1. Position 370. G → A | Confirmed |
| CPHL_A | *ppe 2* | nsS1. Position 419. A → G | Confirmed |
| CPHL_A | *ppe 2* | nsS3. Position 1292. G → A | Confirmed |
| CPHL_A | *ppe 61* | FS1. Position 796. 5 bp insertion. | Confirmed |
| CPHL_A | *ppe 43* | FS1. Position 448 – 542. 5 bp deletion. | Confirmed |
| CPHL_A | *ppe 8* | nsS2. Position 1240. T → G | Confirmed |
| CPHL_A | *ppe 14* | nsS2. Position 878. C → T | Confirmed |
| CPHL_A | *ppe 13* | FS4/5. Poly C/A region | **False.** Whole genome sequence indicates 10 x C. Our results show 11 x C. |
| CPHL_A | *pe 12* | nsS2. Position 649. C → T | Confirmed |
| CPHL_A | *pe 3* | sS1. Position 39. G → C | Confirmed |
| K85 | *ppe 4* | sS1. Position 460. C → T | Confirmed |
| K85 | *ppe 20* | nsS5. Position 1445. C → T | Confirmed |
| K85 | *ppe 29* | nsS2. Position 439. G → T | Confirmed |
| K85 | *ppe 29* | nsS3. Position 731. C → A | Confirmed |
| K85 | *ppe 8* | nsS13. Position 8484. C → A | Confirmed |
| K85 | *ppe 35* | nsS2. Position 1960. C → A | Confirmed |
| K85 | *ppe 13* | FS4/5. Poly C/A region | Confirmed |
| K85 | *pe 8* | nsS1. Position 511. G → A | Confirmed |
| K85 | *pe 8* | sS2. Position 810. C → T | Confirmed |
| K85 | *pe 4* | nsS4. Position 1108. C → T | Confirmed |
| T17 | *ppe 20* | nsS4. Position 1415. C → T | Confirmed |
| T17 | *ppe 65* | nsS2. Position 1066. G → A | Confirmed |
| T17 | *ppe 26* | nsS3. Position 823. G → A | Confirmed |
| T17 | *ppe28* | FS1. Position 169 – 213. 45 bp deletion/44 bp insertion | **False.** Normal sequence confirmed |
| T17 | *ppe 13* | FS4/5. Poly C/A region | Confirmed |
| T92 | *ppe 36* | nsS1. Position 539. A → C | Confirmed |
| T92 | *ppe 45* | sS1. Position 1227. G → A | **False.** Normal sequence confirmed |
| T92 | *ppe 12* | FS1. Position 1125. 1 bp deletion | **False.** Normal sequence confirmed |
| T92 | *ppe 6* | nsS9. Position 9412. A → G | Confirmed |
| T92 | *ppe 6* | nsS10. Position 9464. G → A | Confirmed |
| T92 | *ppe 64* | FS1. Position 757. 1 bp insertion | Confirmed |
| T92 | *ppe 13* | FS4/5. Poly C/A region | Confirmed |
