# Supplementary material for: Comparative Analysis of Mycobacterium tuberculosis pe and ppe Genes Reveals High Sequence Variation and an Apparent Absence of Selective Constraints
Source: PLoS One. 2012 Apr 4;7(4):e30593. doi: 10.1371/journal.pone.0030593 (PMC3319526; doi:10.1371/journal.pone.0030593)
Supplement: Table S5 — (DOCX) [file pone.0030593.s005.docx]

**Table S5.**

| **Gene** | **Sequence variations** | **Position** | **Genetic change** | **Amino acid change** | **Number**  **of isolates** | **Strain specificity of**  **Variation/Comments** |
| --- | --- | --- | --- | --- | --- | --- |
| ***pe35*** | **nsSNP** | | | | | |
|  | nsS1 | 295 | T → G | Stop → Glu.  1 additional aa added. | 1 | SAWC 1870 |
| ***pe11*** | None detected | | | | | |
| ***pe3*** | **nsSNPs** | | | | | |
|  | nsS1 | 40 | G → A | Ala → Thr | 1 | SAWC 4302 |
|  | nsS2 | 523 | T → C | Ser → Pro | 4 | CAS family |
|  | nsS3 | 824 | T → C | Met → Thr | 1 | SAWC 1109 |
|  | nsS4 | 1102 | C → T | Arg → Trp | 1 | LCC “5 bander” (SAWC 1162). |
|  | **sSNP** | | | | | |
|  | sS1 | 183 | A → G | Glu | 1 | SAWC 1956 |
| ***pe_pgrs16*** | **In-frame deletions** | | | | | |
|  | D1 | 865 - 951 | 87 bp deletion |  | 1 | SAWC 2901 |
|  | D2^b^ | 1017 - 1082 | 66 bp deletion |  | 3 | Unrelated PGG2 strains SAWC 198, 233 and 716. Convergent. |
|  | D3 | 1024 - 1641 | 618 bp deletion |  | 1 | SAWC 1109 |
|  | D4 | 1035 - 1100 | 66 bp deletion |  | 2 | EAI isolates SAWC 1659 and 2493. |
|  | D5 | 1219 - 1230 | 12 bp deletion |  | 1 | SAWC 2073 |
|  | D6 | 1267 - 1866 | 600 bp deletion |  | 2 | EAI isolates SAWC 1659 and 2493. |
|  | D7 | 1395 - 1475 | 81 bp deletion |  | 1 | SAWC 2088 |
|  | D8^b^ | 1579 - 1830 | 252 bp deletion |  | 1 | SAWC 2185 |
|  | **Frameshifts** | | | | | |
|  | FS1^b^ | 1969 | 1 bp insertion | Premature stop | 13 | All LCC and Haarlem  (including pre-Haarlem and  Haarlem-like) Isolates. |
|  | **nsSNPs** | | | | | |
|  | nsS1^b^ | 881 | A → G | Asn → Ser | 7 | All Haarlem group except pre-Haarlem isolate SAWC 1748. |
|  | **sSNPs** | | | | | |
|  | sS1 | 555 | C → T | Thr | 1 | SAWC 2803 |
|  | sS2 | 2637 | C → T | Thr | 1 | SAWC 4981 |
|  | sS3 | 1227 | C → A | Gly | 1 | SAWC 2525 |
| ***pe_pgrs18*** | **In-frame deletions** | | | | | |
|  | D1* | 961 - 1086 | 66 bp deletion |  | 1 | SAWC 1127 |
|  | **In-frame insertion/deletion** | | | | | |
|  | Indel1* | 295 | 114 bp deletion/  126 bp insertion |  | 12 | All LCC & Haarlem isolates. Previously reported as the 12/40 polymorphism [30]. |
|  | **Frameshifts** | | | | | |
|  | FS1 | 48 | 19 bp insertion | Premature stop | 1 | SAWC 2701 |
|  | **nsSNPs** | | | | | |
|  | nsS1* | 129 | C → A | His → Gln | 20 | SAWC 1659, 2493, 4981, 2088, 3656, 2576, 2525, 1733,3100, 861, 716, 1748, 1127, 103, 1645,  1841, 2185, 239, 1956. |
|  | nsS2* | 217 | G → C | Glu → Gln | 9 | SAWC 2701, 2076, 3656, 2576, 2525, 1815, 1733, 3100, 861. |
|  | nsS3* | 247 | A → G | Ser → Gly | 8 | SAWC 4981, 3656, 2576,  2525, 1815, 1733, 3100, 861. |
|  | nsS4* | 791 | C → T | Ala → Val | 1 | SAWC 1815 |
|  | nsS5 | 808 | G → A | Ala → Thr | 12 | All PGG1 isolates. |
|  | nsS6 | 962 | T → C | Val → Ala | 4 | CAS family |
|  | nsS7 | 1088 | A → G | Asn → Thr | 1 | SAWC 2525. |
|  | **sSNPs** | | | | | |
|  | sS1* | 54 | G → A | Ala | 20 | SAWC 1659, 4981, 2088,2701, 2076, 3656, 2576, 2525, 1815, 1733, 3100, 716, 1748, 1127, 103, 1645, 1841, 2185, 1956, 4302. |
|  | sS2* | 153 | G → C | Ala | 21 | SAWC 1659, 2493, 4981, 2088, 2701, 2076, 3656, 2576, 2525, 1815, 1733, 3100, 198, 716, 1748, 1127, 103, 1645, 1841, 2185, 1956. |
|  | sS3* | 213 | C → A | Tyr | 9 | SAWC 2701, 2076, 3656, 2576, 2525, 1815, 1733, 3100, 861. |
|  | sS4* | 462 | G → C | Gly | 3 | EAI family. |
|  | sS5* | 471 | T → G | Gly | 7 | All EAI & CAS isolates. |
|  | sS6* | 765 | C → A | Thr | 1 | SAWC 1815 |
|  | sS7 | 774 | T → C | Gly | 12 | All PGG1 isolates. |
|  | sS8 | 1116 | C → T | Pro | 1 | SAWC 1645 |
|  | sS9 | 1263 | G → C | Gly | 1 | SAWC 239 |
| ***pe_pgrs26*** | **In-frame deletions** | | | | | |
|  | D1^b^ | 567 - 584 | 18 bp deletion |  | 3 | Beijing family. |
|  | D2 ^b^ | 842 - 898 | 57 bp deletion |  | 3 | PGG3 isolates SAWC 239, 1608,1109. |
|  | D3 ^b^ | 1004 - 1060 | 57 bp deletion |  | 5 | LAM isolates SAWC 3656, 2576, 2525, 1733 and 3100. |
|  | D4 | 1015 - 1074 | 60 bp deletion |  | 1 | SAWC 198. |
|  | D5 | 1061 - 1108 | 48 bp deletion |  | 1 | SAWC 1109 |
|  | D6 | 1195 - 1203 | 9 bp deletion |  | 1 | SAWC 4981 |
|  | D7 | 1127 - 1174 | 48 bp deletion |  | 1 | SAWC 2493 |
|  | **nsSNPs** | | | | | |
|  | nsS1^b^ | 368 | G → C | Gly → Ala | 1 | SAWC 4981 |
|  | nsS2^b^ | 686 | G → A | Gly → Asp | 2 | SAWC 861 and 1162 (4 and 5 banders). |
|  | nsS3^b^ | 707 | A → G | Asp → Gly | 10 | All PGG1 isolates. |
| ***pe_pgrs33*** | **In-frame deletions** | | | | | |
|  | D1 | 640 - 648 | 9 bp deletion |  | 2 | SAWC 1430 and 1595. |
|  | D2 | 684 - 728 | 45 bp deletion |  | 1 | SAWC 861 |
|  | D3^a^ | 710 - 816 | 75 bp deletion. Result of 107 bp deletion plus 32 bp insertion. |  | 1 | SAWC 1109. 32 bp insertion is a  duplication of nucleotides 611 – 642. |
|  | D4 | 772 - 813 | 42 bp deletion |  | 4 | CAS family. |
|  | **In-frame insertions** | | | | | |
|  | I4^a^ | 1240 | 9 bp insertion |  |  | All PGG3 and PGG2 (LAM) isolates. |
|  | **Frameshift** | | | | | |
|  | FS1 | 554 - 555 | 2 bp deletion | Premature stop | 1 | SAWC 2701 |
|  | FS2^a^ | 1014 | 1 bp deletion | Premature stop | 5 | EAI and LCC ( 2 and 3 bander) specific. Possible convergent mutation. |
|  | **nsSNPs** | | | | | |
|  | nsS1 | 235 | G → A | Ala → Thr | 1 | SAWC 2666 |
|  | nsS2 | 320 | C → T | Ala → Phe | 1 | SAWC 2493 |
|  | nsS3 | 689 | G → C | Gly → Ala | 1 | SAWC 233 |
|  | nsS4^a^ | 701 | T → C | Val → Ala | 1 | SAWC 1109 |
|  | nsS5^a^ | 1166 | G → A | Gly → Asp | 1 | SAWC 2073 |
|  | nsS6 | 1367 | G → A | Gly → Asp | 1 | SAWC 1733 |
|  | **sSNPs** | | | | | |
|  | sS1 | 285 | G → A | Ala | 1 | SAWC 2240 |
|  | sS2^a^ | 717 | C → T | Ala | 19 | All PGG3 and PGG2 (LAM) isolates. |
| ***pe_pgrs62*** | **nsSNP** | | | | | |
|  | nsS1 | 462 | G → C | Gln → His | 3 | EAI family. |
|  | nsS2 | 880 | G → A | Asp → Asn | 2 | SAWC 1659 and 2493. |
|  | **sSNP** | | | | | |
|  | sS1 | 786 | G → C | Leu | 1 | SAWC 2076 |
|  | sS2 | 1099 | T → C | Leu | 1 | SAWC 1430 |
| ***ppe68*** | **nsSNP** | | | | | |
|  | nsS1 | 86 | C → T | Ala → Val | 4 | All true Haarlem isolates. |
|  | nsS2 | 685 | G → C | Val → Leu | 3 | EAI family. |
| ***ppe2*** | **nsSNP** | | | | | |
|  | nsS1 | 887 | T → C | Leu → Pro | 4 | CAS family. |
|  | **sSNP** | | | | | |
|  | sS1 | 834 | G → A | Ala | 1 | SAWC 103. |
|  | sS2 | 1236 | C → T | Thr | 3 | EAI family. |
| ***ppe44*** | **nsSNP** | | | | | |
|  | nsS1 | 31 | A → G | Asn → Asp | 1 | SAWC 2525 |
|  | nsS2 | 176 | G → T | Gly → Val | 3 | EAI family. |
|  | nsS3 | 581 | T → C | Phe → Ser | 11 | All PGG1 isolates. |
|  | nsS4 | 713 | C → T | Pro → Leu | 1 | SAWC 974 |
|  | **sSNP** | | | | | |
|  | sS1 | 1060 | A → C | Gly | 1 | SAWC 2240 |
| ***ppe10*** | **In-frame deletion** | | | | | |
|  | D1 | ? | 15 bp deletion |  | 1 | SAWC 2803 |
|  | **Frameshift** | | | | | |
|  | FS1 | ? | 14 bp insertion | Premature stop | 1 | SAWC 2185 |
|  | **nsSNP** | | | | | |
|  | nsS1 | 440 | G → C | Trp → Ser | 4 | CAS family. |
| ***ppe42*** | **sSNP** | | | | | |
|  | sS1 | 637 | C → A | Arg | 3 | SAWC 861 and 1162. |
| ***ppe62*** | **Frameshift** | | | | | |
|  | FS1 | 323 | 1 bp isertion | Premature stop | 6 | PGG3 isolates SAWC 1608, 1870, 1901, 1956, 239 and 1109. |
|  | **nsSNP** | | | | | |
|  | nsS1 | 478 | G → A | Ala → Thr | 1 | SAWC 861 |
|  | nsS2 | 1026 | C → A | Ser → Arg | 8 | All Haarlem group isolates. |
|  | **sSNP** | | | | | |
|  | sS1 | 1329 | G → A | Thr | 1 | SAWC 198 |
