# Supplementary material for: Comparative Analysis of Mycobacterium tuberculosis pe and ppe Genes Reveals High Sequence Variation and an Apparent Absence of Selective Constraints
Source: PLoS One. 2012 Apr 4;7(4):e30593. doi: 10.1371/journal.pone.0030593 (PMC3319526; doi:10.1371/journal.pone.0030593)
Supplement: Table S6 — (DOCX) [file pone.0030593.s006.docx]

**Table S6.**

| **Gene** | **Primer sequences** | **Tm (°C)** | **Product size (bp)** |
| --- | --- | --- | --- |
| *pe35* | pe35F: CGAGCCTCCAGAAGAAGTGTT  pe35R: GAAAGCGTCTGCCATCCC | 55 | 622 |
| *pe11* | pe11F: TTGTGACCGAAGCCGTTAT  pe11R: CGTAGCTCAGCTCGTTGGA | 55 | 558 |
| *pe3* | pe3F: CTGATGAGTGGCGGTCTCG  pe3R: GTACATCGGGATGACGCTG | 63 | 1724 |
| *pe_pgrs16* | pe_pgrs16F: TGGGATCGGCGACGCTACCAACCAA  pe_pgrs16R: GCCCGCTGCAGACGCCCCTTC  pe_pgrsseqF: GGCAACGGCGGGCTGCTATTCG (internal sequencing primer)  pe_pgrs16seqR: GAGGCCGATGTTGCCGTTGTG (internal sequencing primer) | 64 | 2891 [20] |
| *pe_pgrs18* | pe_pgrs18F: GCAGGGATCGTCCGAATAAA  pe_pgrs 18R: CGGTCACGCCCACAAGGTG  pe_pgrs 18seqF: CAGGGCCTACCCTTTGAG (internal sequencing primer) | 55 | 1561 |
| *pe_pgrs26* | pe_pgrs 26F: AGACCTGCATTTGCAGCAGTC  pe_pgrs26R: GCTGTTCGTTACCGGCATCTG  pe_pgrs26seqF: CCGTCCCCGCCAGCCCT (internal sequencing primer) | 55 | 1797 [20] |
| *pe_pgrs33* | pe_pgrs33F: CTACGGTAACCCGTTCATCCC  pe_pgrs33R: GCGCCCGCCGAAGTGTAAG  PGRS33seqF: GACGGCGGAATCTTGATC (internal sequencing primer) | 55 | 1649 [18] |
| *pe_pgrs62* | pe_pgrs62F: TCACATTCTGGCTTTTGCG  pe_pgrs62R: CCCATGCGTGGCTACGAC | 55 | 1780 |
| *ppe68* | ppe68F: ATGAGGTCTCCGCCCAAGC  ppe68R: TCTTTACCTTCCTCGCCAAAA | 55 | 1383 |
| *ppe2* | ppe2F: ACGCTCCAAACCTTGTCTAGC  ppe2R: GCGTCGCTTGTCGTACCC | 55 | 1828 |
| *ppe44* | ppe44F: TGGCGAGCGTGGTGCCTACGCTGGC  ppe44R: GGGTTAGCGCAATGTGGC  ppe44seqF: GACCTTGATGGAATAATGG (internal sequencing primer) | 62 | 1292 |
| *ppe10* | ppe10F: GCGTCGGTGCTTGCCATA  ppe10R: CAGGTAAGCGAACGGGTAGTC |  | 1757 |
| *ppe42* | ppe42F: GGGCGAAGTTTGACGGAA  ppe42R: TTGCAACTAGGCGAGCTGTT | 60 | 2017 |
| *ppe62* | ppe62F: CCGTAACGCATTGACGACACC  ppe62R: CAACGAACTGGGACTG | 60 | 1936 |
